# Bioinspired, Mitochondria‐Targeted Single‐Atom Nanozyme Enhances Bone Regeneration by Reprogramming Stem Cell Energy Metabolism​

**DOI:** 10.1002/adma.202522108

**Published:** 2026-04-07

**Authors:** Yuwen Wang, Xinzhi Liang, Tiandi Xiong, Zheng Zhong, Ning Zhang, Boguang Yang, Dong Li, Qiongjiao Zeng, Xian Chen, Yiting Lei, Shangsi Chen, Chao Zheng, Liu Yang, Wei Huang, Rocky S. Tuan, Denghui Xie, Zhong Alan Li

**Affiliations:** ^1^ Department of Biomedical Engineering Faculty of Engineering The Chinese University of Hong Kong Shatin Hong Kong China; ^2^ Department of Orthopedic Surgery Center For Orthopedic Surgery The Third Affiliated Hospital, Southern Medical University Guangdong Provincial Key Laboratory of Bone and Joint Degeneration Diseases Guangzhou China; ^3^ InnoHK Center for Neuromusculoskeletal Restorative Medicine Hong Kong Science Park Hong Kong China; ^4^ Shun Hing Institute of Advanced Engineering The Chinese University of Hong Kong Hong Kong China; ^5^ Department of Orthopedic Surgery The First Affiliated Hospital of Chongqing Medical University Chongqing China; ^6^ Institute of Orthopedic Surgery Xijing Hospital Fourth Military Medical University Xi'an China; ^7^ Institute For Tissue Engineering and Regenerative Medicine School of Biomedical Sciences The Chinese University of Hong Kong Shatin Hong Kong China

**Keywords:** bone regeneration, mitochondrial energy metabolism, nanoparticles, osteogenic differentiation, stem cells

## Abstract

Normal mitochondrial function in stem cells is essential for effective bone regeneration, with mitochondrial complex IV (cytochrome *c* oxidase, CcO) playing a crucial role in sustaining electron transport chain activity and ATP synthesis. To address mitochondrial dysfunction associated with bone defects, we developed a dendritic mesoporous silica nanoparticle (DMSN)‐based, CcO‐mimetic nanozyme, named triphenylphosphonium (TPP)‐DMSN‐Fe/Cu. The nanozyme incorporated iron and copper single atoms to mimic the catalytic center of CcO and is modified with the mitochondria‐targeting agent TPP. In vitro, TPP‐DMSN‐Fe/Cu nanozymes colocalized with mitochondria and enhanced mitochondrial function, effectively regulating cellular energy metabolism and promoting stem cell osteogenesis. In vivo, TPP‐DMSN‐Fe/Cu nanozymes resulted in significantly enhanced bone regeneration compared to the control, resulting in a 177% increase in bone volume and a 12% increase in mineral density at critical‐sized bone defects in rats after 4 weeks of treatment. Taken together, these findings demonstrate that bioinspired, mitochondria‐targeting TPP‐DMSN‐Fe/Cu nanozymes hold strong promise for accelerating bone regeneration via regulating cellular energy metabolism.

## Graphical abstract

A bioinspired nanozyme—triphenylphosphonium (TPP)‐dendritic mesoporous silica nanoparticle (DMSN)‐Fe/Cu—mimics mitochondrial complex IV and targets mitochondria to regulate cellular energy metabolism. This approach markedly boosts bone regeneration in vivo, as demonstrated by enhanced bone volume and mineral density in critical‐sized bone defects rat models. The study opens new avenues for mitochondria‐targeted therapies in bone repair.

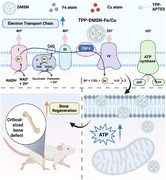

## 1 Introduction

Successful bone regeneration relies on the high energy output of stem cell mitochondria, particularly during the initial stage and matrix synthesis phases, when substantial ATP is required [1]. Mitochondrial complex IV, also called cytochrome *c* oxidase (CcO), is the terminal and essential enzyme of the electron transport chain (ETC) and plays a central role in this energy generation process [2]. Utilizing oxygen as the terminal electron acceptor, CcO supports ATP synthase to generate the majority of cellular ATP through oxidative phosphorylation (OXPHOS).

Studies have demonstrated that alterations in mitochondrial function, particularly impaired electron transport mediated by Complex IV, reduce ATP production and disrupt redox balance, ultimately limiting stem cell survival and new bone formation [3, 4, 5, 6]. Specifically, the disruption of electron transfer from cytochrome *c* (Cyt *c*) to CcO results in inadequate energy generation [4]. This impact is especially pronounced in critical‐sized bone defects (CSBDs), where the demand for efficient energy production is significantly increased. However, the central role of mitochondrial energy metabolism has not been sufficiently addressed in conventional bone regeneration strategies [7, 8, 9].

Mitochondria, the primary source of ATP production, play a pivotal role in regulating stem cell fate during bone regeneration [10, 11]. As stem cells undergo osteogenic differentiation, they increase mitochondrial biogenesis to meet rising energy demands [12]. Notably, undifferentiated stem cells, including bone marrow mesenchymal stem cells [13], embryonic stem cells [14], and hematopoietic stem cells [15], exhibit a stronger reliance on glycolysis than on OXPHOS and fatty acid oxidation (FAO). However, as differentiation progresses, there is a shift in metabolic patterns characterized by increased OXPHOS and FAO and a decline in glycolytic activity [16, 17]. This shift in metabolic priorities is a hallmark of cellular differentiation. Particularly, fatty acid β‐oxidation not only supplies energy but also provides the necessary substrates for OXPHOS [18, 19].

Given the accumulation of cellular reactive oxygen species (ROS) in bone defects, ROS‐scavenging biomaterials have garnered significant interest. Examples include polyphenols, artificial selenoenzymes, hydrogen‐containing materials, catalase (CAT), polydopamine, and chiral biomaterials [20, 21, 22, 23, 24]. While these materials are effective in scavenging ROS, most do not address the underlying issues of mitochondrial dysfunction and compromised cellular energy metabolism [25, 26]. Although various nanozymes have been developed as ROS‐scavenging biomaterials by virtue of their high catalytic activity, stability, and versatility, few are capable of regulating mitochondrial function and energy metabolism to promote tissue regeneration [27]. Therefore, there is a strong need for innovative nanozyme‐based therapeutics that not only enhance mitochondrial function but also create a pro‐regeneration environment to support robust bone regeneration.

**FIGURE 1:**
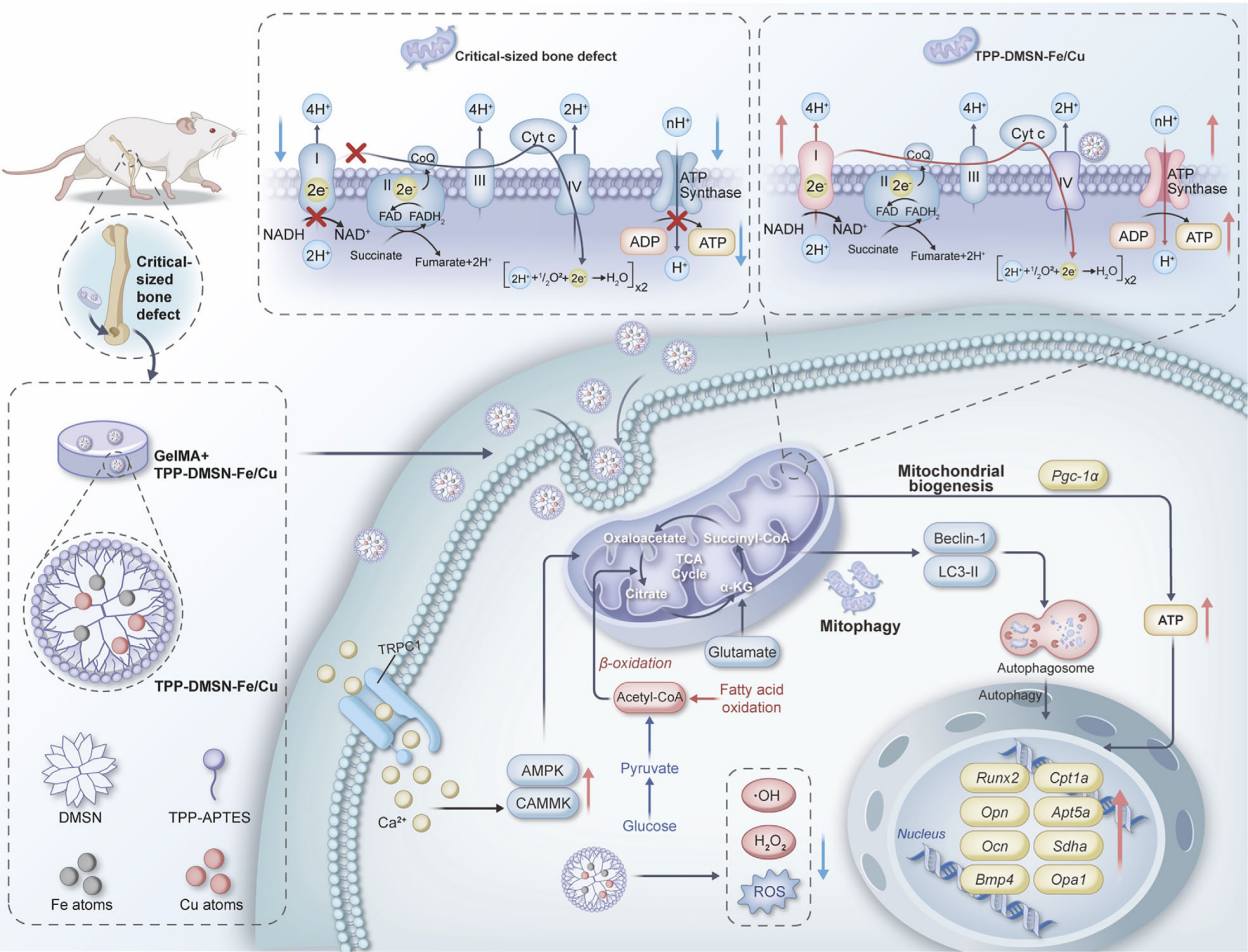
Schematic diagram illustrating the ability of TPP‐DMSN‐Fe/Cu nanozymes to improve mitochondrial function, regulate cellular energy metabolism, and promote osteogenic differentiation of stem cells.

Herein, we developed a CcO‐inspired, mitochondria‐targeted nanozyme, triphenylphosphine (TPP)‐dendritic mesoporous silica nanoparticle (DMSN)‐Fe/Cu, to enhance mitochondrial energy metabolism in stem cells and facilitate CSBD repair. This nanozyme is constructed from DMSN, surface modified with TPP for targeting mitochondria, and loaded with dispersed Fe and Cu atoms to mimic the catalytic centers of CcO (Figure 1). This design was inspired by the impaired electron transfer from Cyt *c* to CcO in dysfunctional mitochondria, which contributes to redox imbalance and energetic failure [28]. The CcO‐like catalytic function of the nanozyme enhanced ETC activity, while TPP ensured efficient mitochondrial delivery. This bioinspired single‐atom nanozyme supported OXPHOS and FAO in stem cells, resulting in improved ATP generation and a cellular environment conducive to bone regeneration. In a rat CSBD model, TPP‐DMSN‐Fe/Cu nanozyme effectively accelerated bone regeneration.

## 2 Results

### 2.1 Synthesis and Characterization of Bioinspired, CcO‐Mimetic TPP‐DMSN‐Fe/Cu Nanozyme

**FIGURE 2:**
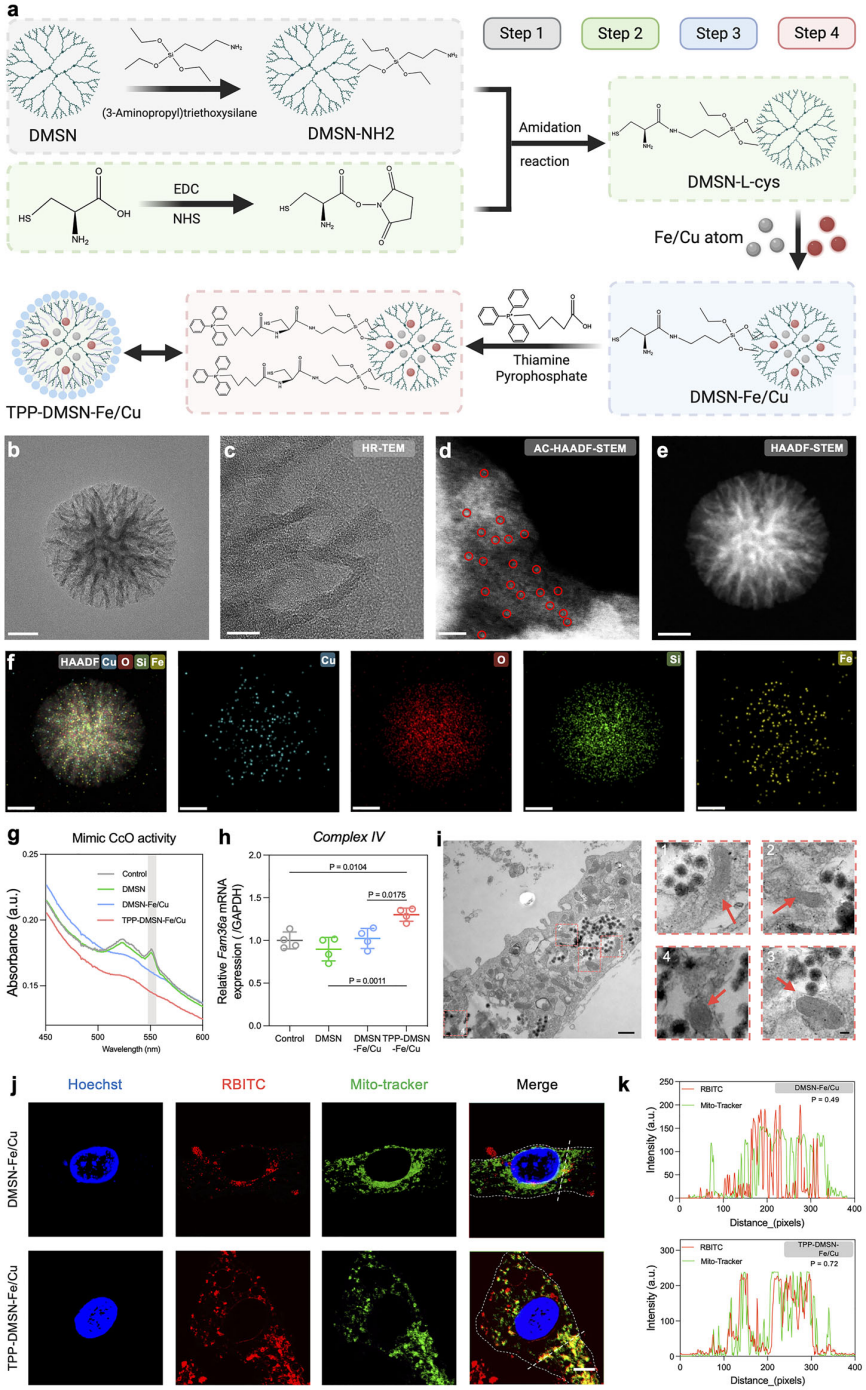
Synthesis and characterization of TPP‐DMSN‐Fe/Cu nanozymes. (a) Schematic of the synthesis process of TPP‐DMSN‐Fe/Cu nanozyme. (b, c) TEM images of the synthesized nanozyme. Scale bar = 50 nm and 10 nm. (d) AC‐HAADF‐STEM image of TPP‐DMSN‐Fe/Cu nanozyme, with single iron atoms marked by red circles. Scale bar = 2 nm. (e) HAADF‐STEM image showing the dendritic mesoporous structure of the synthesized nanozyme. Scale bar = 50 nm. (f) EDS elemental mapping confirming homogeneous distribution of Cu, Fe, Si, and O in TPP‐DMSN‐Fe/Cu nanozyme. Scale bars = 50 nm. (g) UV–vis absorbance spectra of Cyt *c* after reacting with different nanoparticles for 60 min. (h) TPP‐DMSN‐Fe/Cu nanozyme upregulated the expression of *Fam36a* (Cytochrome *c* Oxidase Assembly Factor COX20) gene. Data are presented as mean ± s.d., *n* = 4 biologically independent samples, by one‐way ANOVA with Tukey’s post hoc test. The *P* value is noted. (i) Bio‐TEM images showing the mitochondria‐targeting ability of TPP‐DMSN‐Fe/Cu nanozymes after treating C3H/10T1/2 cells for 4 h (red arrows indicate mitochondria). Scale bar = 500 nm (left image) and 100 nm (magnified views on the right). (j) Representative confocal images showing that the TPP‐modified nanozymes had a higher level of colocalization with C3H/10T1/2 cells compared to nanozymes without TPP modification (blue, nucleus; red, RBITC‐nanoparticles; green, Mito‐tracker). Scale bar = 10 µm. (k) Quantitative analysis of fluorescence intensity along the white dotted lines in j. P = Pearson's correlation coefficient.

DMSNs, known to possess high biocompatibility [29], were synthesized via a one‐step reaction and exhibited uniform spherical morphology with well‐defined mesopores, as confirmed by transmission electron microscopy (TEM) (Figure S1). Subsequent functionalization steps involved sequential loading of Fe/Cu and covalent conjugation of mitochondria‐targeting TPP through amidation, yielding the final TPP‐DMSN‐Fe/Cu nanozyme (Figure 2a–c). In aberration‐corrected high‐angle dark‐field scanning TEM (AC‐HAADF‐STEM) images, the isolated bright spots confirm the atomically dispersed distribution of iron and copper atoms (Figure 2d). HAADF‐STEM imaging revealed a dendritic architecture with radially oriented mesopores (Figure 2e), which probably facilitated efficient loading and stable dispersion of single‐atom catalytic centers. Energy‐dispersive X‐ray spectroscopy (EDS) elemental mapping confirmed the homogeneous distribution of Cu, Fe, Si, and O within the composite structure (Figure 2f). Notably, our TPP‐DMSN‐Fe/Cu nanozyme exhibited CcO‐like catalytic activities, as evidenced by the significantly reduced α‐band absorption (550 nm) of ferrous Cyt *c* in the presence of Fe/Cu‐containing nanozymes (DMSN‐Fe/Cu and TPP‐DMSN‐Fe/Cu), indicating oxidation of Cyt *c* to the oxidized form (Figure 2g) [30]. In contrast, DMSN lacks this catalytic ability, confirming the functions of Fe/Cu‐integrated nanozymes as a CcO analog. The physicochemical properties of the nanozyme were also systematically examined. Zeta potential measurements demonstrated a shift from negative surface charge toward positive surface charge after TPP conjugation (Figure S2a), with an average surface charge value of 34.7 mV, indicating colloidal stability (absolute values >30 mV) [31]. In addition, the use of Fourier‐transform infrared (FTIR) spectroscopy further corroborated the successful synthesis of the nanozyme, with the presence of characteristic Si–O–Si stretching vibrations (410, 755, and 1019 cm^−1^) and a C = O absorption band (1607 cm^−1^) from L‐cysteine‐mediated amidation being observed (Figure S2b). Furthermore, nitrogen adsorption‐desorption isotherms revealed a high specific surface area (592.8 m^2^ g^−1^) and nanoporous structure (Figure S2c) for TPP‐DMSN‐Fe/Cu nanozyme, which are advantageous for Fe/Cu loading and catalytic activities.

We used C3H/10T1/2 cells, a widely used stem cell line for studying bone regeneration [32], for in vitro evaluation of the nanozymes. The biocompatibility of TPP‐DMSN‐Fe/Cu nanozyme was assessed using the cell counting kit 8 (CCK8) assay and Live/Dead staining. Following a 24 h incubation of the cells with different nanoparticles at varying concentrations, the half‐maximum inhibitory concentration (IC_50_) values of DMSN, DMSN‐Fe/Cu, and TPP‐DMSN‐Fe/Cu nanozymes were determined to be 91.71, 73.69 and 107.01 µg/mL, respectively, by fitting the dose‐response curves (Figure S3a,b). Live/Dead staining images also agreed with the CCK8 results (Figure S3c,d). Based on these fidings, we selected a concentration of 10 µg/mL for our subsequent in vitro experiments. Compared to the control group (no nanoparticle treatment), TPP‐DMSN‐Fe/Cu nanozymes resulted in a 30% increase in the expression level of *Fam36a* (also known as *Cox20*), which is essential for the assembly and maturation of mitochondrial complex IV [33]. (Figure 2h). This finding suggests that TPP‐DMSN‐Fe/Cu nanozyme treatment effectively enhanced the activity of mitochondrial complex IV. Subsequent investigation utilizing Bio‐TEM revealed that after 4 h of incubation, TPP modified nanozymes aggregated in proximity to the mitochondria within C3H/10T1/2 cells, with direct contact with the mitochondrial surface observed (Figure 2i; Figure S4). Fluorescence imaging of Rhodamine B isothiocyanate (RBITC)‐labelled nanozyme and MitoTracker‐stained mitochondria showed a higher level of colocalization between TPP‐modified nanozymes and mitochondria than between non‐targeted DMSN‐Fe/Cu nanozymes and mitochondria (Figure 2j,k). To quantify the degree of co‐localization, we performed a Pearson's correlation coefficient analysis for the confocal images. The DMSN‐Fe/Cu group yielded a Pearson's coefficient of 0.49, indicating moderate overlap with mitochondria. In contrast, the TPP‐modified group exhibited a higher coefficient of 0.72, confirming enhanced mitochondrial targeting efficiency. These results are consistent with previous reports demonstrating chemical‐mediated mitochondrial targeting [34, 35], while the non‐targeted group showed limited colocalization, validating the specificity of our targeting strategy. Collectively, these findings proved the CcO‐like catalytic function and mitochondrial targeting ability of the TPP‐DMSN‐Fe/Cu nanozyme.

### 2.2 Bioinspired TPP‐DMSN‐Fe/Cu Nanozymes Enhanced Mitochondrial Function and Energy Metabolism of Stem Cells

**FIGURE 3:**
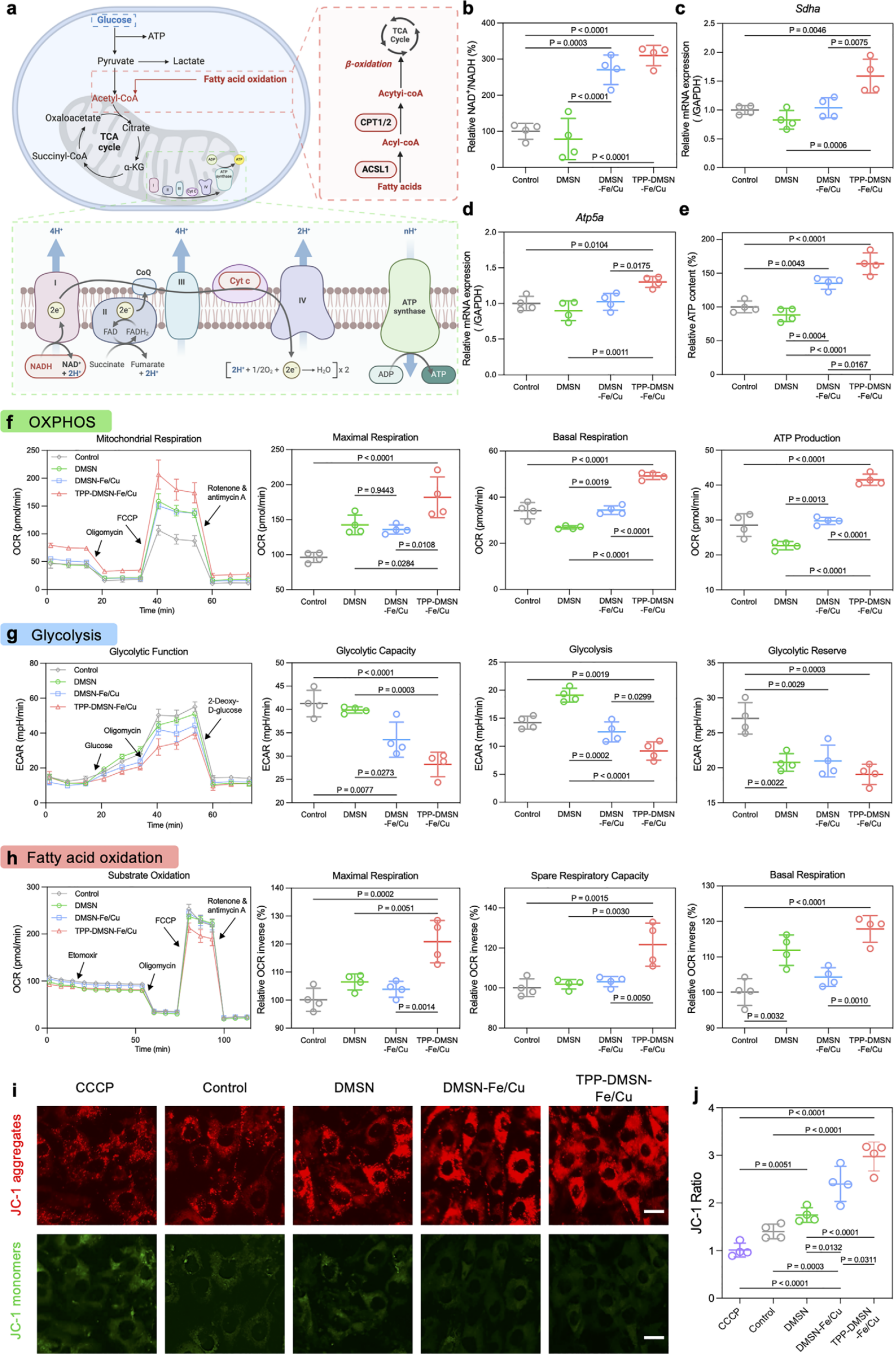
TPP‐DMSN‐Fe/Cu nanozymes improved mitochondrial function and regulated energy metabolism in stem cells. (a) Schematic diagram of TPP‐DMSN‐Fe/Cu nanozyme‐induced enhancement of OXPHOS, FAO, and TCA cycle and the changes in related target metabolites. (b–e) TPP‐DMSN‐Fe/Cu nanozymes treatment resulted in the highest levels of b NAD^+^/NADH, (c) *Sdha* and (d) *Atp5a* mRNA, and (e) ATP production in stem cells. (f) Real‐time OCRs during stem cell mitochondrial stress tests and semi‐quantitative analysis of basal respiration, maximal respiration, and ATP production. (g) Real‐time ECARs of stem cells during the glycolytic stress test and semi‐quantitative analysis of glycolysis, glycolytic capacity, and glycolytic reserve. (h) Real‐time OCRs of stem cells during the substrate oxidation test and semi‐quantitative analysis of maximal respiration, spare respiratory capacity, and basal respiration. (i) Representative image of JC‐1 aggregates and monomers and j corresponding quantitative analysis indicating the highest mitochondrial membrane potential in cells treated by TPP‐DMSN‐Fe/Cu nanozymes for 7 days in osteogenic medium. Scale bar = 25 µm. Data are presented as mean ± s.d., *n* = 4 biologically independent samples, by one‐way ANOVA with Tukey's post hoc test. The *p*‐value is noted.

We then verified the capability of our bioinspired TPP‐DMSN‐Fe/Cu nanozymes in augmenting mitochondrial function and modulating cellular energy metabolism (Figure 3a). OXPHOS relies on an ETC consisting of five enzyme complexes (*I*–*V*), in which complexes I–IV establish a proton gradient to drive complex V to synthesize ATP [36]. Lipid metabolism also contributes to ATP production. FAO converts lipids to acetyl‐CoA, which fuels the TCA [37]. Moreover, previous studies have demonstrated that osteoblasts can acquire energy from fatty acids, a process imperative for bone tissue formation [38]. While designed to activate mitochondrial complex IV, the TPP‐DMSN‐Fe/Cu nanozyme was found to also activate other ETC complexes, including complexes I, II, and V, thereby augmenting the entire ETC. Specifically, the nanozyme promoted the conversion of NADH to NAD+, a key function of complex I (Figure 3b). Gene expression analysis demonstrated significant upregulation of *Sdha*, which encodes a subunit of complex II, and *Atp5a*, which encodes a subunit of complex V, in TPP‐DMSN‐Fe/Cu nanozyme‐treated stem cells (Figure 3c,d). [39, 40] In line with these findings, ATP luminescence assays revealed a 63.9% increase in cellular ATP levels in the TPP‐DMSN‐Fe/Cu nanozyme group in comparison to the control group (Figure 3e), suggesting enhanced mitochondrial ETC function.

It has been demonstrated that fatty acid β‐oxidation increases dramatically as osteoblasts mature in vitro, and that anabolic Wnt signaling via LRP5 promotes FAO [41]. To evaluate potential nanozyme‐induced metabolic shift, mitochondrial respiration and glycolysis were analyzed using a Seahorse Extracellular Flux Analyzer. Initially, real‐time oxygen consumption rate (OCR) was recorded after the continuous addition of oligomycin, Carbonyl cyanide‐4 (trifluoromethoxy) phenylhydrazone (FCCP), and rotenone as well as antimycin A. It was found that with TPP‐DMSN‐Fe/Cu nanozyme treatment, the basal respiration, maximum respiration, mitochondrial ATP production, non‐mitochondrial oxygen consumption, and spare respiratory capacity were increased by 44%, 89%, 45%, 138%, and 114%, respectively, compared with the control group (Figure 3f; Figure S5). These results indicate that these nanozymes could effectively enhance mitochondrial function. Subsequently, we monitored the changes in extracellular acidification rate (ECAR) after sequential addition of glucose, oligomycin, and 2‐deoxy‐D‐glucose (2‐DG). Compared with the other groups, the glycolysis, glycolytic capacity, and glycolytic reserve of the TPP‐DMSN‐Fe/Cu group were significantly reduced (Figure 3g).

To elucidate metabolic reprogramming, OCR was also monitored after sequential inhibition of FAO (via etomoxir), ATP synthase (via oligomycin), and mitochondrial respiration (via rotenone/antimycin A). TPP‐DMSN‐Fe/Cu‐treated cells demonstrated a reduction in both basal and maximal respiration in comparison to the control group (Figure 3h). The observed decrease in OCR after etomoxir treatment (FAO inhibition) in nanozyme‐treated cells suggests an increased dependence on FAO for energy production. These findings suggest that TPP‐DMSN‐Fe/Cu nanozymes reprogram cellular metabolism by enhancing FAO. Additionally, the TPP‐DMSN‐Fe/Cu nanozyme‐treated group showed increased mitochondrial membrane potential, indicating enhanced cellular energy metabolism (Figure 3i,j; Figure S6). Collectively, these results demonstrated that the bioinspired, CcO‐mimetic TPP‐DMSN‐Fe/Cu nanozymes augmented mitochondrial energy metabolism through OXPHOS‐ and FAO‐driven bioenergetics in stem cells, potentially promoting their osteogenic differentiation.

### 2.3 Transcriptomic Analysis Revealed TPP‐DMSN‐Fe/Cu Nanozyme‐Induced Promotion of Energy Metabolism and Bone Regeneration‐Related Signaling Pathways in Stem Cells

**FIGURE 4:**
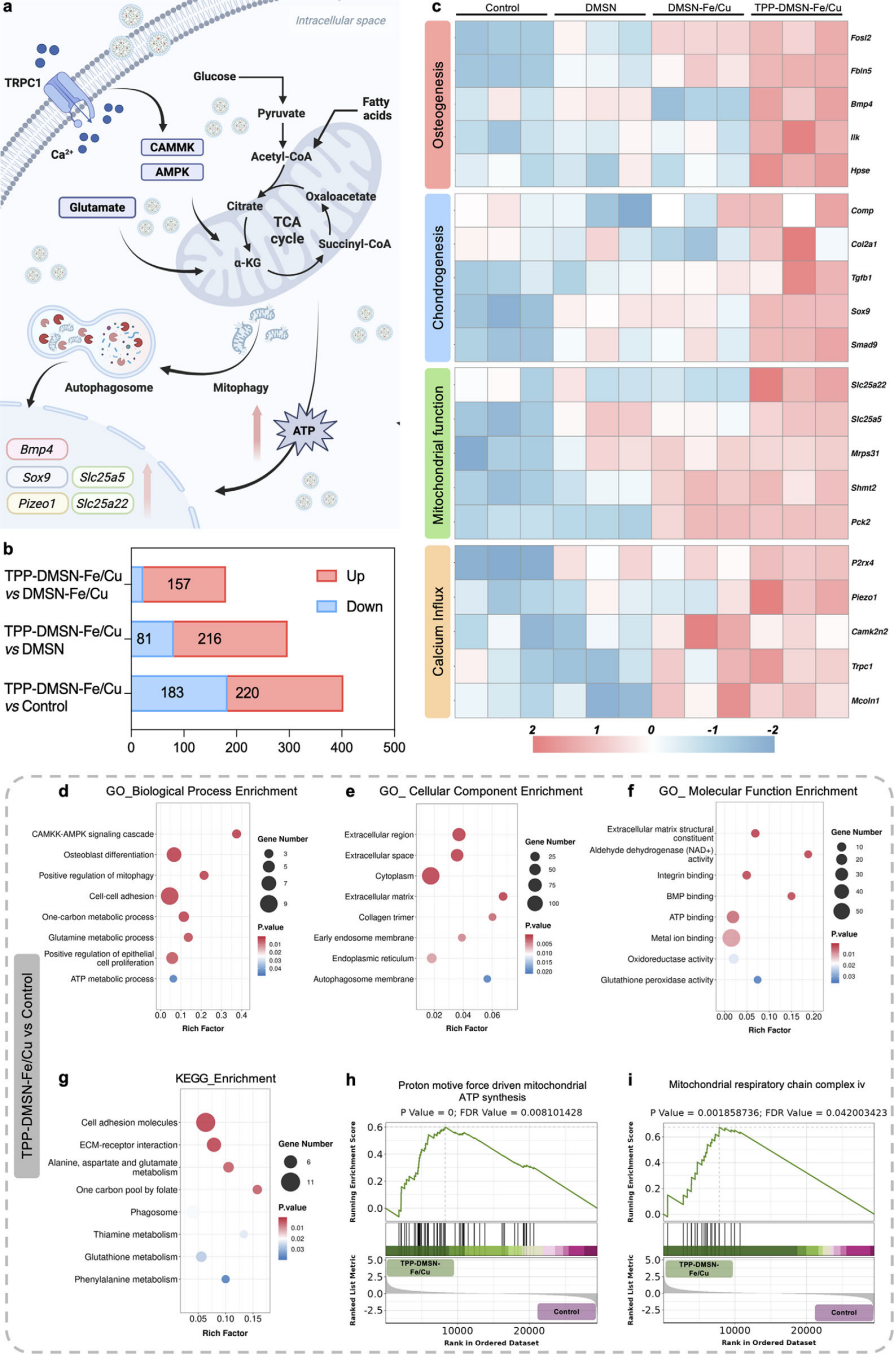
Transcriptome analysis revealed that TPP‐DMSN‐Fe/Cu nanozymes reprogramed stem cell metabolism to enhance osteogenesis. (a) Schematic of TPP‐DMSN‐Fe/Cu‐modulated mitochondrial and osteogenic pathways based on RNA sequencing results. (b) Number of differentially expressed genes in TPP‐DMSN‐Fe/Cu‐treated stem cells versus other groups. (c) Heatmap showing upregulated osteogenesis‐, chondrogenesis‐, calcium influx‐, and mitochondrial function‐related genes for DMSN‐Fe/Cu‐treated stem cells. *n* = 3 biologically independent samples. (d–f) Gene Ontology enrichment analysis of biological process (d), cellular component (e), and molecular function (f) for up‐regulated genes in TPP‐DMSN‐Fe/Cu nanozymes. (g) KEGG enrichment analysis showing genes upregulated by TPP‐DMSN‐Fe/Cu treatment. (h, i) GSEA plot showing the enrichment of the gene set of “Proton motive force driven mitochondrial ATP synthesis” and “Mitochondrial respiratory chain complex iv”. The top panels of the figures show the enrichment score (green line). The middle panels show the presence of the target gene in the gene set. The bottom panels show the drop in the ranked gene list for the ranked metric value.

In order to investigate TPP‐DMSN‐Fe/Cu‐induced changes in metabolic and osteogenic gene expression, RNA sequencing was performed. The transcriptomic analysis revealed the key mechanisms underlying the nanozyme's ability to enhance mitochondrial function and osteogenic differentiation (Figure 4a). In comparison with the control group, the DMSN group, and the DMSN‐Fe/Cu‐treated group, the TPP‐DMSN‐Fe/Cu group showed 220, 216, and 157 upregulated genes, respectively (Figure 4b). Of note, the expression levels of *Bmp4*, *Sox9*, *Trpc1*, and *Slc25a22* were found to be significantly increased for the TPP‐DMSN‐Fe/Cu nanozyme group (Figure 4c). Among these genes, the osteogenic marker *Bmp4* is crucial for maintaining matrix synthesis and promote the osteogenic differentiation of stem cells [42]. Chondrogenesis, the process of cartilage formation, generates cartilage that can serve as the initial skeletal structure and as a template for endochondral ossification [43]. As a chondrogenic marker, Sox9 is the primary transcription factor necessary for the differentiation of stem cells into chondrocytes and subsequent cartilage formation [44]. Beyond its role in the initiation of chondrogenesis, *Sox9* has also been shown to regulate stem cell metabolism by modulating FAO [45]. It has been established that *Trpc1*, in its capacity as a functional channel for calcium ion influx, is capable of regulating calcium ion influx [46]. This, in turn, activates CAMMK/AMPK signaling pathway and promotes mitochondrial biogenesis. The mitochondrial glutamate transporter *Slc25a22* is a pivotal transporter that supplies carbon substrates to the TCA cycle via glutathione synthesis [47].

Gene Ontology (GO) enrichment analysis (Figure S7) of biological processes revealed activation of pathways linked to mitochondrial function and osteogenesis, including CAMKK‐AMPK signaling cascade, ATP metabolic process, glutamine metabolic process, and positive regulation of osteoblast proliferation (Figure 4d). These findings consistently support the nanozyme's capacity to enhance mitophagy, a selective autophagy process critical for eliminating dysfunctional mitochondria, which is closely linked to bone regeneration. Furthermore, cellular component analysis revealed an enrichment in ECM categories, including extracellular region, space, matrix, and collagen trimers, suggesting enhanced collagen biosynthesis (Figure 4e). Additionally, molecular function analysis identified enhanced NAD^+^ activity, oxidoreductase activity, and glutathione peroxidase activity for the TPP‐DMSN‐Fe/Cu nanozyme group, thereby supporting the nanozyme's role in mitochondrial optimization and osteogenesis (Figure 4f). Furthermore, Kyoto Encyclopedia of Genes and Genomes (KEGG) pathway analysis highlighted enriched metabolic pathways pivotal to bone formation, including alanine/aspartate/glutamate metabolism, glutathione metabolism, and phagosome activity, for the TPP‐DMSN‐Fe/Cu group (Figure 4g). Gene Set Enrichment Analysis (GSEA) further corroborated these findings, revealing increased mitochondrial respiratory chain complex IV activity, autophagy regulation, glutamate transport, and ATP synthesis for the TPP‐DMSN‐Fe/Cu nanozyme‐treated cells (Figure 4h,i; Figure S8).

GO enrichment analysis (Figure S9) comparing the TPP‐DMSN‐Fe/Cu nanozyme‐treated cell group with the DMSN‐Fe/Cu nanozyme‐treated group also revealed TPP‐DMSN‐Fe/Cu nanozyme‐induced activation of pathways associated with mitochondrial function and osteogenesis. These include positive regulation of the CAMKK‐AMPK signaling cascade, mitochondrial autophagy, ATP metabolism, and ossification. Furthermore, GSEA analysis revealed upregulated Cyt *c* release from mitochondria in TPP‐DMSN‐Fe/Cu nanozyme‐treated cells compared to the DMSN‐Fe/Cu nanozyme‐treated group. Collectively, these results demonstrate that TPP‐DMSN‐Fe/Cu nanozymes drive osteogenesis by augmenting mitochondrial metabolism and activating bone regeneration‐related signaling pathways.

### 2.4 Functional Validation of TPP‐DMSN‐Fe/Cu nanozyme‐Enhanced Osteogenic Differentiation and Mitochondrial Metabolic Activation

**FIGURE 5:**
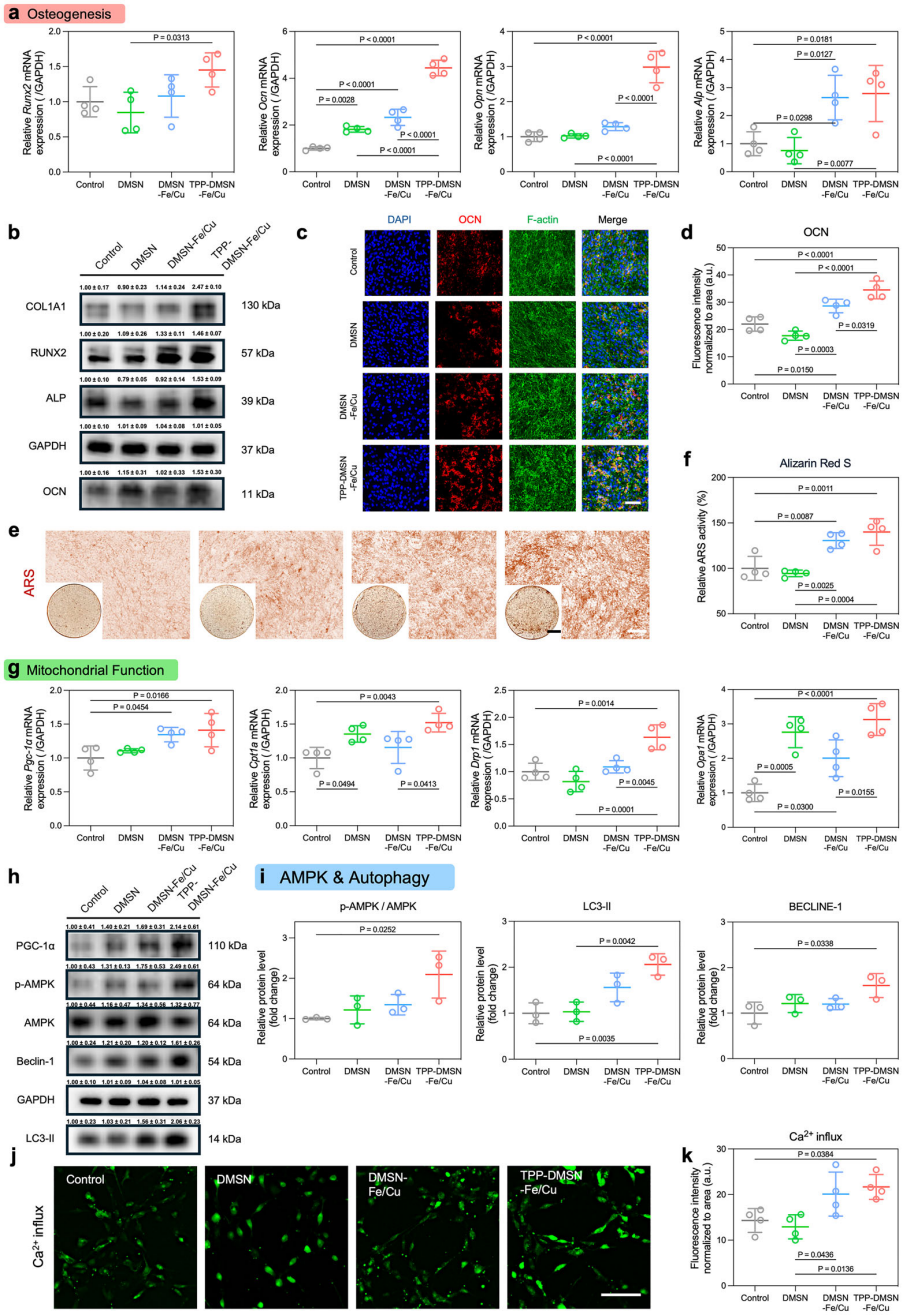
TPP‐DMSN‐Fe/Cu nanozymes enhanced osteogenic differentiation and mitochondrial function in stem cells. (a) mRNA levels of osteogenic gene expression: *Runx2*, *Opn, Ocn, and Alp*. Data are presented as mean ± s.d., *n* = 4 biologically independent samples, by one‐way ANOVA with Tukey's post hoc test. The *p*‐value is noted. (b) Western blot bands for COL1A1, RUNX2, ALP, and OCN (the numbers above each band represent normalized mean band intensity ± s.d., *n* = 3 biologically independent samples). (c) Immunofluorescence staining of OCN and d quantification of fluorescent intensity. Data are presented as mean ± s.d., *n* = 4 biologically independent samples, by one‐way ANOVA with Tukey's post hoc test. The *p* value is noted. Scale bar = 100 µm. (e) ARS staining of stem cells and f corresponding quantitative analysis after 14 days of nanoparticle treatment. Data are presented as mean ± s.d., *n* = 4 biologically independent samples, by one‐way ANOVA with Tukey's post hoc test. The *p* value is noted. Scale bar = 2 mm and 100 µm. (g) mRNA levels of mitochondrial gene expression: *Pgc‐1α*, *Cpt1a*, *Drp1, and Opa1*. Data are presented as mean ± s.d., *n* = 4 biologically independent samples, by one‐way ANOVA with Tukey's post hoc test. The *p*‐value is noted. (h) Western blot bands for PGC‐1α, p‐AMPK, AMPK, Beclin‐1 and LC3‐II (the numbers above each band represent normalized mean band intensity ± s.d., *n* = 3 biologically independent samples). (i) Quantitative analysis of p‐AMPK/ AMPK, Beclin‐1 and LC3‐II protein expression. (j) Intracellular calcium ion levels and k corresponding quantification. Data are presented as mean ± s.d., *n* = 4 biologically independent samples, by one‐way ANOVA with Tukey's post hoc test. The *p*‐value is noted.

In addition to evaluating TPP‐DMSN‐Fe/Cu nanozyme‐enhanced osteogenesis by transcriptome analysis, we further validated the osteogenic properties of the nanozymes by various other methods. These findings were first confirmed by PCR‐revealed mRNA expression levels. Compared with the control, the transcriptional levels of runt‐related transcription factor 2 *(Runx2)*, osteocalcin *(Ocn)*, osteopontin *(Opn)*, *Alkaline phosphatases (Alp), and collagen type I alpha 1 (Col1a1)* were increased by 1.45, 4.44, 2.99, 2.79, and 2.05 folds, respectively, for the TPP‐DMSN‐Fe/Cu nanozyme group (Figure 5a; Figure S10). At the protein level, TPP‐DMSN‐Fe/Cu nanozyme treatment led to the most prominent upregulation of key osteogenic markers, with COL1A1, RUNX2, ALP, and OCN increased by 147%, 46%, 53%, and 60%, respectively (Figure 5b; Figure S11). Immunofluorescence staining further corroborated these findings, demonstrating heightened expression levels of osteogenic markers (OCN, COL1A1, and RUNX2) and augmented mineralization in stem cells (Figure 5c,d; Figures S12 and S13). ALP activity is an early marker of bone formation. ALP activity was found to be significantly increased after 7 days of nanozyme treatment, with the TPP‐DMSN‐Fe/Cu nanozyme group demonstrating the highest ALP activity (Figure S14). Consistently, Alizarin Red S (ARS) staining revealed the highest level of mineralization for the TPP‐DMSN‐Fe/Cu nanozyme group (Figure 5e,f). These results underscore the capacity of nanozymes to amplify both early‐ and late‐stage osteogenic pathways. It is also worthy of note that genes associated with mitochondrial function was significantly upregulated after TPP‐DMSN‐Fe/Cu nanozymes treatment. In comparison with the control group, the expression levels of *Pgc‐1α*, *Cpt1a*, *Drp1*, and *Opa1* increased by 41%, 52%, 63%, and 213%, respectively, for the TPP‐DMSN‐Fe/Cu nanozyme group (Figure 5g). Among these markers, *Pgc*‐*1α* is involved in the coordination of mitochondrial biogenesis and oxidative metabolism [48], and *Cpt1a* plays a regulatory role in fatty acid uptake for energy production [49]. The expression of mRNA associated with mitochondrial fusion (*Opa1*) and fission (*Drp1*) was evaluated [50, 51]. The results suggest that the TPP‐DMSN‐Fe/Cu nanozymes significantly increased the expression of genes associated with mitochondrial fusion and fission, resulting in a more active dynamic equilibrium in stem cell mitochondria.

KEGG and GO biological process enrichment (Figure 4d, g) suggests that CAMKK‐AMPK signal cascade and autophagy are key mediators linking mitochondrial energy regulation and osteoblast differentiation. These findings are consistent with the mechanisms reported in previous studies on stem cell differentiation [52, 53, 54]. Furthermore, as the primary energy source for cellular activity, the status and number of mitochondria are critical for the repair of damaged tissue [55]. The regulation of mitochondrial biogenesis is a multifaceted process, with PGC‐1α serving as the primary regulatory factor [48]. Additionally, AMPK has been shown to regulate PGC‐1α through a process of phosphorylation, thereby enhancing mitochondrial biogenesis [56]. Therefore, we assessed the relative expression of PGC‐1α, AMPK, and activated AMPK (p‐AMPK) in C3H/10T1/2 cells treated with TPP‐DMSN‐Fe/Cu nanozymes. As illustrated in Figure 5h,i and Figure S15, TPP‐DMSN‐Fe/Cu nanozymes significantly increased the protein expression of PGC‐1α (2.14‐fold) and the p‐AMPK/AMPK ratio (2.09‐fold) in comparison to the control group. In accordance with this observation, treatment of C3H/10T1/2 cells with TPP‐DMSN‐Fe/Cu nanozymes resulted in a 61% and 106% increase in Beclin‐1 and LC3‐II protein levels, respectively, thereby confirming the activation of autophagy (Figure 5h,i). It is noteworthy that Beclin‐1 and its binding partners regulate the activity of the Vps34 lipid kinase, which is crucial for autophagy and other membrane transport processes [57]. It has been established through previous studies that the presence of LC3‐II in lipid form serves as an indicator of autophagy‐related structures [58]. Moreover, it has been demonstrated that mitochondrial autophagy is implicated in the regulation of bone metabolism [54, 59]. In addition, Ca^2+^ fluorescence probe experiments demonstrated that the green fluorescence intensity in the TPP‐DMSN‐Fe/Cu nanozyme group was considerably higher than that in the control group (Figure 5j,k). This finding is indicative of elevated intracellular calcium ion concentrations. Consequently, TPP‐DMSN‐Fe/Cu nanozymes create an energy‐rich microenvironment that promotes osteoblast synthesis and mineralization. This multifunctional approach renders TPP‐DMSN‐Fe/Cu nanozymes a promising therapeutic candidate for accelerated bone regeneration.

Given that ROS accumulation disrupts mitochondrial function and impair tissue regeneration [60], we evaluated the nanozyme's ROS‐scavenging potential using radical elimination assays. It was found that Fe/Cu loading in the nanoparticles significantly increased the rates of scavenging DPPH radical, hydroxyl radical (•OH), and hydrogen peroxide (H_2_O_2_) compared with unmodified DMSN (Figures S16 and S17). This robust antioxidant activity positions TPP‐DMSN‐Fe/Cu nanozymes as a potent therapeutic agent for mitigating oxidative stress and restoring redox balance in stem cells, thereby supporting mitochondrial health during osteogenesis. Furthermore, we assessed the intracellular ROS scavenging efficacy using the fluorescent probe 2′,7′‐dichlorodihydrofluorescein diacetate (DCFH‐DA). In H_2_O_2_‐stimulated stem cells, 10 µg/mL nanoparticles significantly reduced ROS levels, with the ROS scavenging rates of the DMSN, DMSN‐Fe/Cu nanozyme and TPP‐DMSN‐Fe/Cu nanozyme groups being 1.16, 1.41 and 1.72 times those of the untreated control group, respectively (Figure S18). The outstanding ROS scavenging activity of TPP‐DMSN‐Fe/Cu can effectively protect stem cells from oxidative damage in an injurious environment. Moreover, compared with the control group, the TPP‐DMSN‐Fe/Cu nanozymes increased the expression level of *Cat* mRNA by 47% (Figure S19), indicating that our bioinspired nanozymes significantly upregulated the antioxidant capacity of stem cells. These properties collectively support their promising potential in bone regeneration applications.

### 2.5 Bioinspired, CcO‐Mimetic TPP‐DMSN‐Fe/Cu Nanozymes Accelerated Bone Regeneration in Rat CSBDs

**FIGURE 6:**
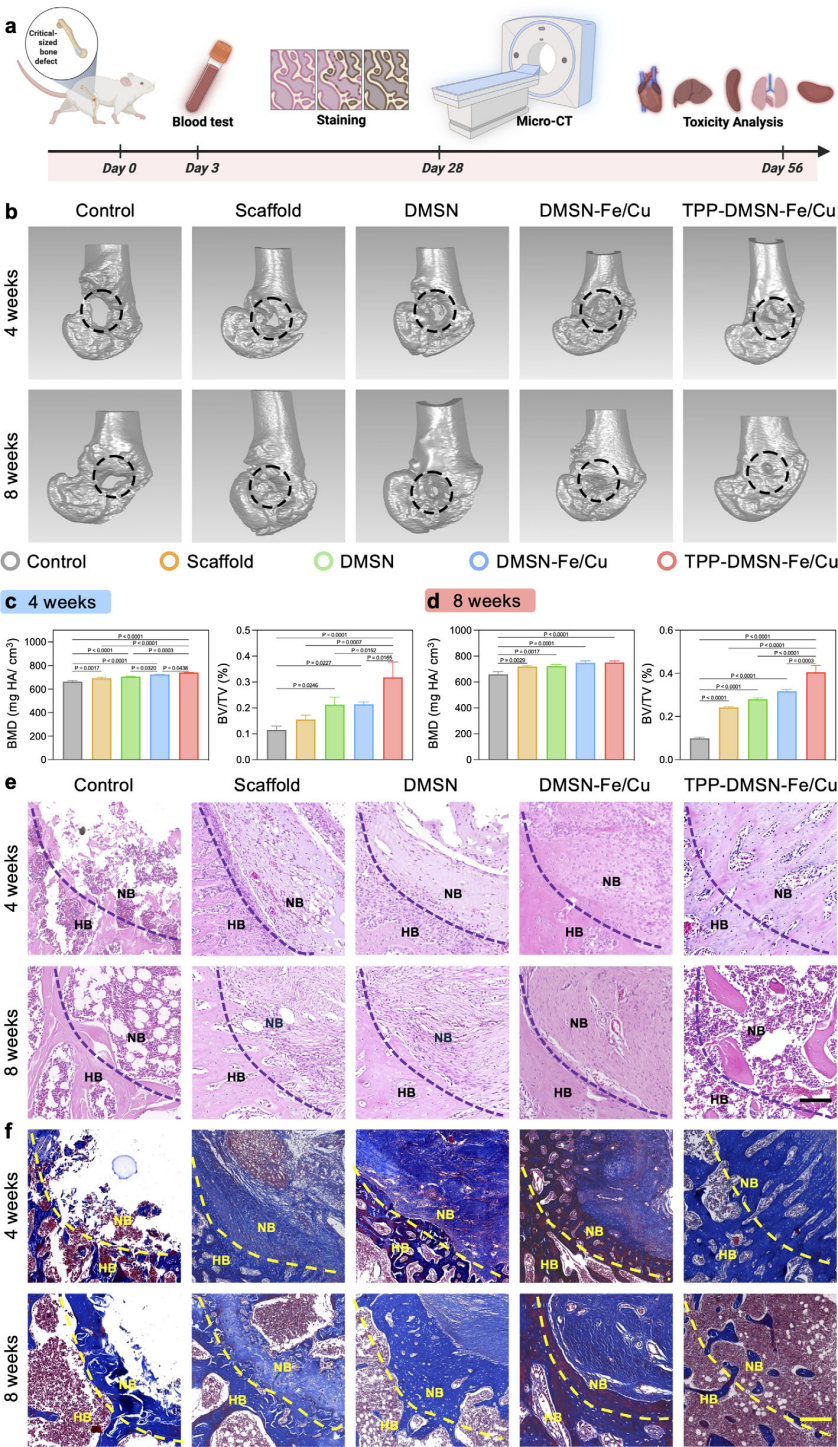
TPP‐DMSN‐Fe/Cu nanozymes accelerated bone regeneration in a rat model of CSBD. (a) Schematic of the experimental timeline. (b) 3D reconstructed micro‐CT scan images after 4 and 8 weeks of scaffold implantation. (c, d) Quantitative micro‐CT analysis of BV/TV and BMD. Data are presented as mean ± s.d., *n* = 3 biologically independent samples, by one‐way ANOVA with Tukey's post hoc test. The *p*‐value is noted. (e) H&E staining and f Masson's trichrome staining images of the bone defect sites at 4 and 8 weeks after the surgery. Scale bar = 100 µm. HB = host bone; NB = newly formed bone.

The effects of TPP‐DMSN‐Fe/Cu nanozymes on bone tissue regeneration in vivo were evaluated using a rat femoral defect model (Figure 6a). The defect sites were implanted with pure gelatin methacryloyl [GelMA, 10% (wt/v)] scaffolds and those modified with 500 µg/mL of DMSN, DMSN‐Fe/Cu, and TPP‐DMSN‐Fe/Cu (denoted GelMA/DMSN scaffolds, GelMA/DMSN‐Fe/Cu scaffolds, and GelMA/TPP‐DMSN‐Fe/Cu scaffolds, respectively). Bone defect specimens were harvested from the femora of Sprague Dawley (SD) rats after 4 and 8 weeks of treatment and evaluated using micro‐CT. As demonstrated in Figure 6b, different amounts of newly regenerated bone were observed in the defect areas of all experimental groups following 4 weeks of treatment. After another four weeks, an increased amount of new bone was observed for all groups. Furthermore, we analyzed the bone volume fraction (BV/TV) and bone mineral density (BMD) of the newly regenerated bone tissues. In comparison with the control group, the BV/TV values for the GelMA/TPP‐DMSN‐Fe/Cu group were significantly higher at both four and eight weeks. At Week 4, the average BV/TV for the GelMA/TPP‐DMSN‐Fe/Cu group was found to be 2.77, 2.05, 1.50, and 1.49 times higher than that for the control, GelMA, GelMA/DMSN, and GelMA/TPP‐DMSN‐Fe/Cu groups, respectively (Figure 6c). At Week 8, these fold changes in BV/TV were further increased to 4.10, 1.67, 1.45, and 1.28, respectively (Figure 6d). Additionally, compared to the control, the scaffold groups with/without nanoparticle addition all showed significantly higher BMD values, with the largest increment observed for the GelMA/TPP‐DMSN‐Fe/Cu group. Furthermore, hematoxylin and eosin (H&E) staining and Masson's trichrome staining were performed for histological analysis of the regenerated bone (Figure 6e,f). At both 4 and 8 weeks, the area and density of new bone for the GelMA/TPP‐DMSN‐Fe/Cu group were greater than those in the control group and the GelMA group. The GelMA/TPP‐DMSN‐Fe/Cu group consistently demonstrated superior defect repair capabilities to the control group.

**FIGURE 7:**
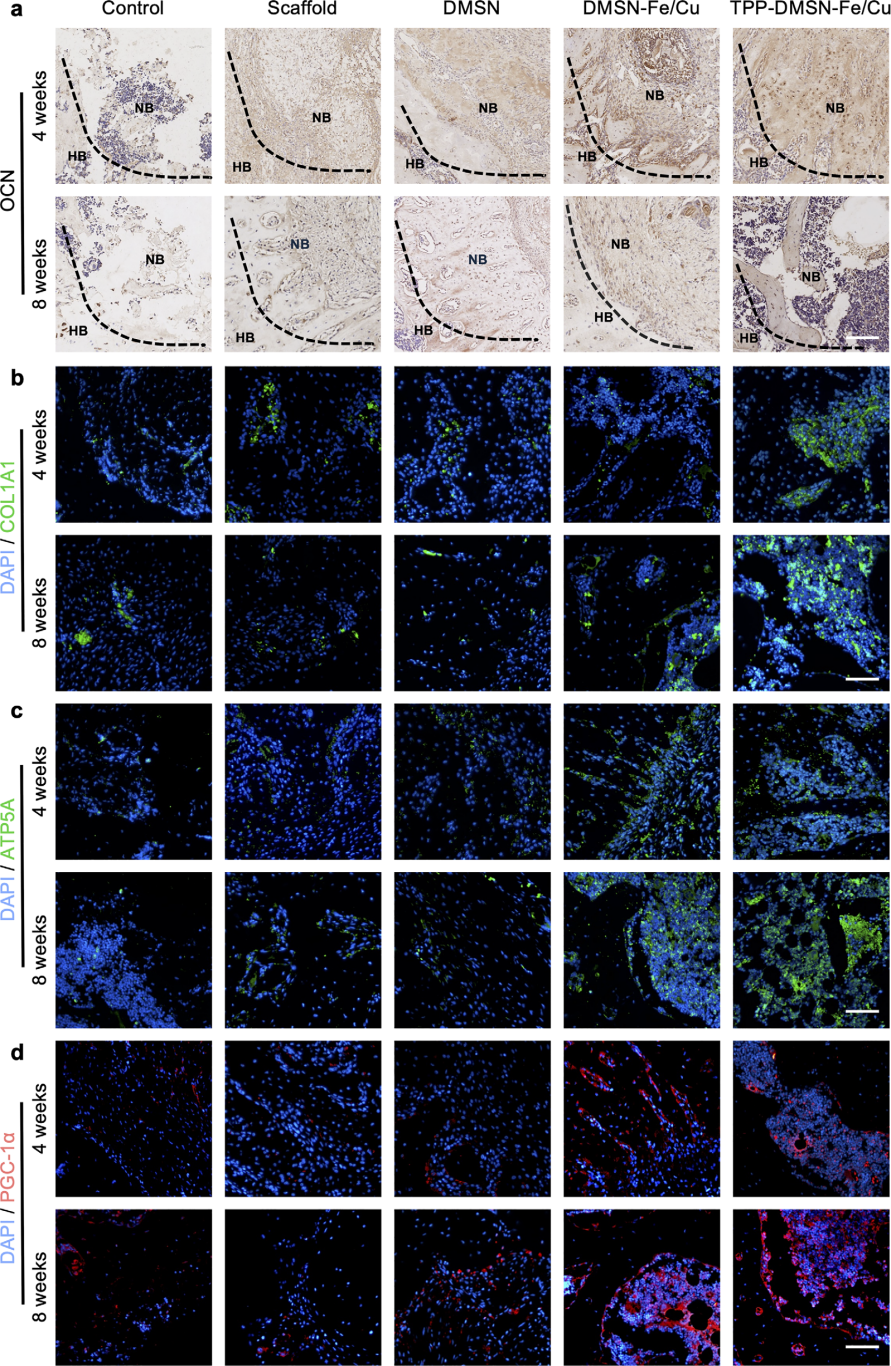
TPP‐DMSN‐Fe/Cu nanozymes upregulated the expression of markers related to osteogenesis and energy metabolism in vivo. (a) OCN IHC staining images for the femoral bone defect at 4 and 8 weeks. Immunofluorescence staining images for b COL1A1, c ATP5A, and d PGC‐1α in the femoral bone defect area at 4 and 8 weeks. Scale bar = 100 µm.

In order to further investigate the processes of osteoblast differentiation and mitochondrial biogenesis during bone regeneration, immunohistochemical (IHC) staining for OCN and immunofluorescence staining for COL1A1, PGC‐1α, and ATP5A were performed. The number of OCN‐positive cells was found to be significantly higher in the GelMA/TPP‐DMSN‐Fe/Cu group than in the other four groups (Figure 7a; Figure S20). Similarly, the COL1A1 expression levels were the highest in the GelMA/TPP‐DMSN‐Fe/Cu group, with the control and GelMA groups showing the lowest levels of COL1A1 among all groups (Figure 7b; Figure S21). These results indicate that TPP‐DMSN‐Fe/Cu nanozymes effectively promoted osteogenic differentiation and bone regeneration. Furthermore, compared with other groups, the expression of PGC‐1α, a mitochondrial biogenesis marker (Figure 7c; Figure S22), and ATP5A, an ATP production marker (Figure 7d; Figure S23), was found to be significantly increased in the GelMA/TPP‐DMSN‐Fe/Cu group at both 4 and 8 weeks. These findings suggest that the application of TPP‐DMSN‐Fe/Cu nanozymes resulted in a larger number of healthy mitochondria, thereby providing a greater energy supply for bone repair. Furthermore, the biocompatibility and biosafety of TPP‐DMSN‐Fe/Cu nanozymes and other nanoparticles were evaluated in vivo. No abnormalities were observed in the heart, liver, spleen, lung, and kidney tissues of rats from any groups, and no obvious difference was observed between the GelMA scaffold only and GelMA/TPP‐DMSN‐Fe/Cu groups, as revealed in H&E staining images (Figures S24 and S25). Moreover, a comprehensive blood analysis revealed comparable conventional blood parameters across all the groups (Figure S26), further supporting the high biosafety of TPP‐DMSN‐Fe/Cu nanozymes.

## 3 Discussion

We developed a bioinspired, mitochondria‐targeted nanozyme that mimics the enzymatic activity of CcO (Complex IV), a pivotal component of the ETC. The single‐atom Fe/Cu was embedded within DMSN, emulating the CcO active sites to achieve efficient redox catalysis. To achieve mitochondrial targeting, the nanozyme surface was functionalized with TPP, a widely used mitochondria‐targeting moiety with a lipophilic cationic structure that facilitates selective enrichment around mitochondria. This design enhanced mitochondrial function, boosted antioxidant capacity, and facilitated OXPHOS and FAO in stem cells, ultimately accelerating defect repair in a rat model of CSBD. By incorporating stepwise controls, including DMSN and DMSN‐Fe/Cu (without TPP), we demonstrated that the CcO‐mimetic DMSN‐Fe/Cu nanozyme only moderately improved cellular energetics. Robust enhancement of mitochondrial metabolism, functional gene expression, and bone regeneration was only achieved with TPP‐mediated mitochondrial localization. These results confirm that both the catalytic capacity of the nanozyme and its targeted mitochondrial delivery are essential to elicit the observed therapeutic effect.

In injured bone microenvironments, a redox imbalance has been demonstrated to drive mitochondrial dysfunction and excessive ROS accumulation, thereby impairing osteogenesis [61]. While ROS‐scavenging biomaterials have demonstrated potential, most of these materials do not regulate mitochondrial homeostasis in a targeted manner [62, 63]. Although emerging mitochondria‐targeted polyphenol/amino acid NPs (e.g., ECGG‐Cys‐NPs, PGA‐Mn‐TP04) [56, 64] enhanced mitochondrial biogenesis and reducing ROS, many of them lack the capacity to effectively drive the metabolic reprogramming required for osteogenesis. Compared to peptide‐based targeting vectors (e.g., TP04) or materials with primarily antioxidant activity (e.g., EGCG‐based or Mn‐based systems), our TPP‐DMSN‐Fe/Cu nanozymes uniquely integrates efficient mitochondrial targeting via the TPP moiety with CcO‐mimicking catalytic activity, supporting energy metabolism beyond antioxidant effects alone. This dual functionality enables more effective reprogramming of stem cell metabolism and bone regeneration compared to previous mitochondria‐targeted platforms.

Unlike previously reported bioinspired nanozymes, our bioinspired nanozyme was specifically designed to mimic the catalytic activity of mitochondrial complex IV, distinguishing it from other bioinspired designs [65, 66, 67]. Rather than focusing solely on antioxidant effects [68, 69], we drew inspiration from both the structure and function of CcO, the terminal enzyme of cellular respiration. By closely emulating this essential enzyme, the TPP‐DMSN‐Fe/Cu nanozymes strengthen mitochondrial electron transport and increases ATP production, thereby boosting overall cellular energy metabolism. While a large number of bioinspired nanozymes possess biomimetic properties, only a small subset have demonstrated targeted mitochondrial delivery or the capacity ot regulate cellular energy metabolism at the level of ETC and compelx IV [70, 71]. This targeted strategy offers clear advantages over systems that merely mitigate oxidative stress, and more effectively supports the metabolic and energetic requirements necessary for bone regeneration.

This study significantly advances mitochondria‐targeted nanotherapeutics that promote FAO‐ and OXPHOS‐driven osteogenic differentiation, supported by integrated transcriptomic and functional validation. Transcriptomic analyses revealed the upregulation of key genes, including, *Pgc‐1α* (amplifying mitochondrial biogenesis) [48], *Slc25a22* (supplying TCA intermediates and glutathione precursors) [47], and *Cpt1a* (enabling fatty acid transport for β‐oxidation) [49], which play critical roles in mitochondrial function and stem cell osteogenesis. These transcriptional changes were corroborated by Seahorse metabolic assays, where nanozyme‐treated cells exhibited increased OXPHOS and FAO, along with reduced glycolysis, establishing a direct mechanistic link between enhanced metabolic gene expression and functional shifts in cellular energy metabolism. Furthermore, the upregulation of mitochondrial markers such as *Sdha* and *Atp5a*, along with regulators of mitochondrial dynamics (*Opa1* and *Drp1*), further supports improved mitochondrial function and homeostasis. Taken together, this multiomics‐to‐function framework robustly demonstrates that our mitochondrial‐targeted nanozyme reprograms stem cell metabolism to drive superior osteogenic and regenerative outcomes.

The observed upregulation of Beclin‐1 and LC3‐II, together with transcriptomic evidence of mitophagy activation, suggests that TPP‐DMSN‐Fe/Cu nanozyme treatment also promotes autophagic processes in stem cells. Enhanced autophagy, especially mitophagy, plays a crucial role in maintaining cellular homeostasis by clearing damaged organelles, such as dysfunctional mitochondria. This renewal of the mitochondrial network ensures a healthy and efficient population of mitochondria to meet the increased energy demands during osteogenic differentiation [59, 72]. Moreover, autophagy has been shown to modulate signaling pathways that favor bone formation and mineralization, while mitophagy can protect stem cells from oxidative stress and enhance their regenerative potential [54].

While the TPP‐DMSN‐Fe/Cu nanozymes hold promising potential in bone regeneration, several questions remain to be answered before the clinical translation of this technology. For example, large‐animal validation of long‐term biocompatibility, biodistribution, and regenerative outcomes should be rigorously assessed. In addition, the mechanisms underlying the body's immune responses to the nanozymes, such as the effects of nanozymes on macrophage polarization, remain to be clarified. To understand the interactions between non‐stem cells and the nanozymes, organ‐on‐a‐chip technology can play an instrumental role in revealing the nanotechnology‐biology interface by, for example, the establishment of a bone defect‐on‐a‐chip system [73]. This system can offer a biomimetic microenvironment, in which a variety of relevant cell types, such as stem cells, osteoblasts, osteoclasts, and endothelial cells, can be cultured in defined compartments under precisely controlled flow and mechanical conditions. By introducing and monitoring selected cell types, this platform allows researchers to systematically decipher the effects of nanozymes on specific cells, including their behaviors (e.g., differentiation, matrix production, angiogenesis, and immune response), and intercellular interactions. The dynamic perfusion and mechanical stimulation within the chip closely resemble the biophysical microenvironment of native bone, ensuring a high level of physiological relevance. This approach aligns with recent policy shifts by regulatory bodies such as the FDA, which support the use of microphysiological systems and other New Approach Methodologies in translational research. We believe a bone defect‐on‐a‐chip model could systematically assess nanozyme effects on distinct cell types within a physiologically relevant context.

We employed C3H/10T1/2 cells, a well‐established murine mesenchymal stem cell line, for our in vitro mechanistic investigations due to their proved osteogenic potential, genetic stability, and experimental reproducibility [74, 75, 76]. This choice enables high consistency and facilitates clear mechanistic interpretation, minimizing the variability often associated with primary cells. Although primary human stem cells would enhance clinical relevance, C3H/10T1/2 cells provide a widely accepted platform for dissecting nanozyme–stem cell interactions. The findings of this research can be extended to primary human cells and in vivo models in future studies. Given the pivotal role of redox imbalance in osteoblast dysfunction and osteoporosis development [28, 77], our TPP‐DMSN‐Fe/Cu nanozymes could also be used for effective osteoporosis treatment, which necessitates both the enhancement of metabolic efficiency in stem cells and the protection of mature osteoblasts from oxidative damage.

## 4 Conclusions

Effective bone regeneration requires robust mitochondrial function in stem cells, with mitochondrial CcO playing a vital role in maintaining ETC activity and driving ATP synthesis. To address CSBD‐associated mitochondrial dysfunction in stem cells, we developed a bioinspired, mitochondria‐targeted TPP‐DMSN‐Fe/Cu nanozyme that mimics CcO activity through the embedded Fe/Cu centers. The surface modification with TPP enabled effective mitochondrial targeting. This CcO‐mimetic nanozyme enhanced mitochondrial function, promoted OXPHOS and FAO in stem cells, and effectively scavenged ROS, thereby creating a microenvironment conducive to osteogenesis. In vivo studies demonstrated significantly accelerated TPP‐DMSN‐Fe/Cu nanozyme‐induced bone regeneration, as evidenced by significant increased bone volume and mineral density. Overall, our study underscores the promising potential of nanozyme‐mediated enhancement of mitochondrial function and energy metabolism for CSBD regeneration.

## 5 Methods

The animal experiments were approved by the Animal Experimental Committee of the Shuiyuntian (Guangdong) Co., Ltd (Approval SYT2024068) and carried out in accordance with the regulations outlined in the National Law on Experimental Animal Utilization (P.R. China).

### 5.1 Chemicals

Triethanolamine (TEA, purity ≥ 98%), Hexadecyltrimethylammonium bromide (CTAB, purity ≥ 99%), Sodium salicylate (NaSal, purity ≥ 99%), Methanol (CH_3_OH, purity ≥ 99%), (5‐carboxypentyl) (triphenyl)phosphonium bromide (TPP, purity ≥ 97%), N‐Hydroxysuccinimide (NHS, purity ≥ 97%), N‐(3‐Dimethylaminopropyl)‐N′‐ethylcarbodiimide hydrochloride (EDC, purity ≥ 99%) were purchased from Sigma–Aldrich. Hydrochloric acid (HCl, 37%) was purchased from Duksan. Tetraethyl orthosilicate (TEOS, purity ≥ 98%), 1,2‐bis(triethoxysilyl) ethane (BTEE, purity ≥ 95%), (3‐Aminopropyl) triethoxysilane (APTES, purity ≥ 99%), Iron sulfate heptahydrate (FeSO_4_‐7H_2_O, purity ≥ 99%), Copper nitrate trihydrate ((Cu (NO_3_)_2_‐3H_2_O, purity ≥ 99%). All aqueous solutions were prepared with deionized water (18.2 MΩ; Millipore).

### 5.2 Synthesis of TPP‐Fe/Cu‐DMSN

- Step 1: DMSN Synthesis.
- The DMSN was synthesized in a procedure that incorporated cationic surfactants CTAB and Nasal. The silicon sources utilized were TEOS and BTEE, and the catalyst employed was TEA. Initially, 0.136 g of TEA was added to 50 mL of water and stirred gently at 80°C for 0.5 h. Subsequently, 760 mg of CTAB and 336 mg of NaSal were added to the solution and stirred at 80°C for an additional hour. The mixture of 8 mL TEOS and 3.2 mL BTEE was then subjected to gentle stirring for 6 h at 80°C at 300 rpm. The resulting mixture was subjected to centrifugation at 10 000 rpm for 10 min and washed once or twice with pure ethanol. Finally, the mixture was subjected to a reflux process with HCL (3 mL): methanol (60 mL) solution for a duration of 2 h at a temperature of 80°C. Subsequently, the mixture was subjected to a centrifugation step and was then washed with ethanol.
- Step 2: DMSN‐ NH_2_ Synthesis.
- In this step of the procedure, 280 mg of DMSN were dissolved in 80 mL of ethanol and sonicated. Subsequently, 7 mL of APTES were added, and the solution was refluxed for 6 h at 70°C. Thereafter, the solution was subjected to centrifugation as previously described.
- Step 3: DMSN‐Fe/Cu Synthesis.
- A solution of 80 mg of DMSN‐NH_2_ in 20 mL of MES buffer at a pH of 6 was prepared initially. This was followed by the activation of 48 mg of EDC and 12.8 mg of NHS for a duration of 30 min. Subsequently, 320 mg of L‐cysteine was incorporated, and the mixture was maintained at a temperature of 37°C for a period of 6 h. The resulting nanoparticles were subjected to a centrifuge process, followed by resuspension in 9 mL of DI water. Subsequently, 1 mL of DI water containing Cu (NO_3_)_2_‐3H_2_O (41 mg) and FeSO_4_‐7H_2_O (100 mg) was added.
- Step 4: TPP‐Fe/Cu‐DMSN
- A mixture of 20 mg of DMSN/L‐Cys/Fe/Cu and 24.552 mg of NHS + 49.288 mg of EDC was dispersed in 10 mL of MES buffer at pH 6.00 and stirred for 2 h at room temperature. Subsequently, 80 mg of TPP was dispersed in 8 mL of DI water and incubated at 37°C for 6 h. The final product was subjected to centrifugation, as previously described.

### 5.3 Cell Culture

C3H/10T1/2, Clone 8 cells were procured from National Collection of Authenticated Cell Cultures (SCSP‐506). For all experiments, cells at passages 11 to 14 were used. Low‐glucose Dulbecco's modified Eagle's medium (DMEM; Gibco) supplemented with 10% fetal bovine serum (FBS) and 1% penicillin‐streptomycin was used as cell culture medium. For osteogenic differentiation, cells were transitioned to high‐glucose DMEM containing 100 nm dexamethasone, 10 mm β‐glycerophosphate, and 50 µg/mL L‐ascorbic acid (Sigma–Aldrich). The differentiation medium was refreshed every 48–72 h to ensure consistent nutrient availability. All culture reagents, unless specified otherwise, were sourced from Gibco.

### 5.4 Cellular Uptake

Nanoparticles were internalized by stem cells were observed using TEM (Hitachi H‐7650; Hitachi, Tokyo, Japan). Cells were cultured in growth medium at an initial density of 5.0 × 10^5^ cells/well in 6‐well plates. When the cells reached 80% confluence, nanozymes were added. After 4 h of incubation, the cells were fixed, dehydrated, embedded, cut, and then observed by TEM. For mitochondrial‐targeted validation, stem cells were incubated in osteogenic differentiation DMEM medium containing nanoparticles (10 µg/mL) for 4 h. Cellular uptake of nanoparticles was observed by confocal microscopy (Leica SP8). Cells and nanoparticles were stained with Mito‐tracker green and RBITC, respectively.

### 5.5 Real‐Time RT‐PCR

The stem cells were seeded into 6‐well plates and allowed to proliferate to 70% confluence. Thereafter, the medium was substituted with osteogenic differentiation medium, and nanoparticles were introduced. After a 7 days incubation, total RNA was extracted using the RNA Extraction Kit (ZYMO). To obtain cDNA, total RNA was reverse transcribed using the cDNA Amplification Kit (YEASEN). The primer sequences employed in this study are listed in (Table S1), obtained from the Primer Library (https://www.ncbi.nlm.nih.gov/gene/) and synthesized by BGI Genomics. The transcript levels of the target genes were calculated using the ΔΔCt method, with GAPDH serving as the housekeeping gene. Cells not treated with nanoparticles were used as the control group.

### 5.6 Western Blotting

Cells were cultured and processed in the same way as for qRT‐PCR assay. On day 7, proteins were extracted using RIPA lysis buffer (Thermo Fisher) and protease and phosphatase inhibitor cocktail, MSF. After BCA (Thermo Fisher) quantification, proteins were loaded into sodium dodecyl sulphate polyacrylamide gels and transferred to Trans‐Blot Turbo Midi 0.2 µm PVDF Transfer Packs (Bio‐Rad). All antibodies used in this study are listed in (Table S2). Band intensity was quantified using ImageJ 1.54 software (NIH, USA).

### 5.7 Calcium^2+^ Influx

Intracellular calcium ion influx imaging was performed using a Leica Thunder imager to record the intensity of the intracellular calcium dye (Fluo‐4 AM, Beyotime). The calcium imaging data obtained were analyzed and visualized using ImageJ 1.54 software (NIH, USA).

### 5.8 Seahorse

Seahorse XFe96 analyzed oxygen consumption rate (OCR) and extracellular acidification rate (ECAR) to assess OXPHOS, glycolysis, and lipid metabolism. Stem cells (1 × 10^4^ cell/mL) were seeded onto a Seahorse XF‐96 plate. For Cell Mito Stress Test, the final concentration of Oligomycin, FCCP, and Rotenone/antimycin is 2, 1, and 0.5 µm. For Glycolysis Stress Test, Glucose, Oligomycin, and 2‐Deoxy‐D‐glucose is 10 mm, 1 µm, and 50 mm. For Substrate Oxidation Stress Test, the final concentration of etomoxir, Oligomycin, FCCP, and Rotenone/antimycin is 4, 1.5, 1.5, and 0.5 µm.

### 5.9 Therapeutic Efficacy of Scaffolds on Critical‐Size Bone Defect Rats Model

Adult SD rats were procured from Guangzhou Shuiyuntian Biotechnology Co. Ltd. The 50 rats (8‐12 weeks, male, 200–220 g) were randomly divided into five groups: empty defect (control), GelMA scaffolds, DMSN + GelMA scaffolds, DMSN‐Fe/Cu + SF/GelMA scaffolds, and TPP‐DMSN‐Fe/Cu + GelMA scaffolds. The nanozyme concentration was determined based on a separate pilot study to achieve sustained nanozyme release in the complex in vivo microenvironment while also ensuring animal health. After the rats were anesthetized with intraperitoneal pentobarbital (35 mg/kg), the femur was exposed via a surgical incision, and a 3‐mm diameter and 3‐mm depth bone defect was created using an electric drill. Scaffolds were then implanted into the defect. Following a 4‐ or 8‐week treatment period, half of the rats were euthanized, and their femora were collected and evaluated using gross observation, micro‐CT scanning, and histological staining.

### 5.10 Histology, Immunohistochemistry, and Immunofluorescence Staining

Tissue samples were decalcified in 10% EDTA‐2Na (Solarbio) at 4°C for 50 days, paraffin‐embedded, and sectioned (5 µm thickness). Sections underwent H&E, Masson's trichrome using commercial kits. All antibodies used in this study are listed in (Table S3). Images intensity was semi‐quantitatively analyzed using  1.54 software (NIH, USA).

### 5.11 Micro‐CT Analysis

Femoral defects were analyzed at 4‐ and 8‐weeks post‐treatment using a Quantum GX2 micro‐CT system at 50 kV and 100 µA (30 µm resolution). A cylindrical volume of interest (VOI; 3 mm diameter, 0.27 mm depth) centered on the defect site was evaluated. BV/TV and BMD were calculated to assess regenerated bone microstructure.

### 5.12 Statistical Analysis

The standard deviation (s.d) of the data in each group is expressed as mean ± s.d. Comparisons between groups were made using one‐way ANOVA followed by Tukey's post hoc test. All statistical analyses were performed with GraphPad Prism 10. At least three biological replicates were used for each experiment.

## Conflicts of Interest

The authors declare no conflicts of interest. Z. A. Li., Y. Wang., and T. Xiong are inventors on a Chinese invention patent application pending approval for the reported single‐atom nanozyme and its preparation method (Application number: 2025110514866).

## Supporting information




**Supporting File**: adma72993‐sup‐0001‐SuppMat.docx.

## Data Availability

The data that support the findings of this study are available from the corresponding author upon reasonable request.
