# Supplementary material for: Bioinspired, Mitochondria‐Targeted Single‐Atom Nanozyme Enhances Bone Regeneration by Reprogramming Stem Cell Energy Metabolism​
Source: Adv Mater. 2026 Apr 7;38(26):e22108. doi: 10.1002/adma.202522108 (PMC13155285; doi:10.1002/adma.202522108)
Supplement: Supplementary file 1 — Supporting File: adma72993‐sup‐0001‐SuppMat.docx. [file ADMA-38-e22108-s001.docx]

Supporting Information

Bioinspired, Mitochondria-targeted Single-atom Nanozyme Enhances Bone Regeneration by Reprogramming Stem Cell Energy Metabolism​

*Yuwen Wang ^1,#^, Xinzhi Liang ^3,#^, Tiandi Xiong ^1,4,#^, Zheng Zhong ^1,3^, Ning Zhang ^1,3^, Boguang Yang ^1,4^, Dong Li ^1,4^,Qiongjiao Zeng ^1,2^, Xian Chen ^1,2^, Yiting Lei ^1,5^, Shangsi Chen ^1,4^, Chao Zheng ^6^, Liu Yang ^6^, Wei Huang ^5^, Rocky S. Tuan ^1,4,7,*^, Denghui Xie ^3,*^, Zhong Alan Li ^1,2,4,7,*^*

1. Department of Biomedical Engineering, Faculty of Engineering, The Chinese University of Hong Kong, Shatin, Hong Kong SAR, China
2. Shun Hing Institute of Advanced Engineering, The Chinese University of Hong Kong, NT, Hong Kong SAR, China
3. Department of Orthopedic Surgery, Center for Orthopedic Surgery, and Guangdong Provincial Key Laboratory of Bone and Joint Degeneration Diseases, The Third Affiliated Hospital of Southern Medical University, Guangzhou, 510630, PR China
4. InnoHK Center for Neuromusculoskeletal Restorative Medicine, Hong Kong Science Park, NT, Hong Kong SAR, P.R. China
5. Department of Orthopedic Surgery, The First Affiliated Hospital of Chongqing Medical University, Chongqing, 400016, China
6. Institute of Orthopedic Surgery, Xijing Hospital, Fourth Military Medical University, Xi’an, 710032, China
7. Institute for Tissue Engineering and Regenerative Medicine, School of Biomedical Sciences, The Chinese University of Hong Kong, Shatin, Hong Kong SAR, China

​

^#^ These authors contributed equally to this work.

^*^ Corresponding authors: tuanr@cuhk.edu.hk (RST); [xiedenghui221122@smu.edu.cn](mailto:xiedenghui221122@smu.edu.cn) (DHX) alanli@cuhk.edu.hk (ZAL).

**This file includes: Figure S1-S26, Table S1-S3**

**S.Ⅰ. Materials and methods**

Characterization of nanoparticles

The Ultraviolet–visible (UV–vis) transmittance spectra were obtained by SpectraMax iD3 plate reader (Molecular Devices, USA). The structural features of nanoparticles were analyzed by transmission electron microscopy (TEM; Hitachi H7700) and elemental analyses were performed by scanning transmission electron microscope (STEM; Talos F200X). Zetasizer (Malvern) was used to test the zeta potential of prepared nanoparticles. FT-IR data was tested by Cary 630 FTIR Spectrometer.

Cell viability

Cell Counting Kit-8 (CCK-8; APExBIO, USA) was used to determine the viability and proliferation of stem cells. Cell viability using cell densities of 1 × 10^4^ cells/well seeded at the bottom of 96-well plates. After the cells reached 80% - 90% confluence, nanoparticles were added. 10% of the total volume of CCK-8 reagent was added to each well. After 120 min of incubation, the optical density of each well was measured at 450 nm using a SpectraMax iD3 microplate spectrophotometer (Molecular Devices, USA).

Alkaline phosphatase (ALP) activity and staining

The stem cells were seeded into 6-well plates and when the cells reached 70% confluence, the growth medium was changed to bone differentiation medium. At the same time, nanoparticles were added. On day 7, the ALP activity of the cells was detected using the ALP Assay Kit (Shanghai Beyotime Biotechnology Institute). Western and IP cell lysates were added at 100 ul per well, and after dilution, 50 μL of supernatant and 50 μL of color development substrate were mixed in a 96-well plate. The plate is then incubated at 37 °C for 10 minutes in the dark before adding 100 μL of termination solution and measuring absorbance at 405 nm. For staining, the stem cells were seeded into 24-well plates at a density of 5×10^4^ cells/well. On day 7, cells were washed by PBS 3 times and fixed by 10% formalin. Subsequently, the BCIP/NBT ALP Staining Kit was used according to the manufacturer's instructions. The ALP staining results were observed using an inverted light microscope (Nikon, ECLIPSE Ts2) and photographed using a digital camera.

Alizarin red staining (ARS) and quantification

The cells were cultured and treated in the same way as ALP staining. On day 14, the cells were washed, fixed and stained with 2% ARS staining solution (Sigma Aldrich). The results of ARS staining were observed and photographed according to the ALP staining procedure. After desorption of the mineral deposits stained with 10% (w/v) cetylpyridinium chloride (Aladdin), the absorbance was measured at a wavelength of 562 nm

RNA sequencing

Total RNA was isolated from stem cells using TRIzol reagent, followed by quantification (Nanodrop ND-1000) and integrity assessment (Agilent Bioanalyzer 2100). Polyadenylated mRNA was enriched via oligo(dT) magnetic bead selection (Thermo Fisher) and fragmented at 94°C for 5 min using a magnesium-based RNA fragmentation kit (New England BioLabs). First-strand cDNA synthesis was performed with reverse transcriptase (Thermo Fisher), and second-strand synthesis was achieved using RNase H and DNA polymerase I (New England BioLabs). Double-stranded DNA libraries were prepared by end-repair with dUTP (Thermo Fisher), adapter ligation, and UDG-mediated excision of the second strand (New England BioLabs). Libraries were amplified via PCR (8 cycles: 98°C denaturation, 60°C annealing, 72°C extension) and size-selected (300 ± 50 bp) using magnetic beads. Sequencing was performed on an Illumina Novaseq 6000 platform by LC-Bio Technologies (Hangzhou, China). RNA-seq data, which can be downloaded from NCBI GEO (GSE324502).

H_2_O_2_ Catalytic Elimination Assay

A mixture of 10 mM H_2_O_2_ and 100 μg/mL nanoparticles in PBS was prepared (2 mL total volume). At hourly intervals over 6 hours, 100 μL of the reaction solution was combined with 100 μL titanium sulphate (Ti (SO₄)₂) solution (32 mg Ti (SO₄)₂ and 0.83 mL H₂SO₄ in 5.33 mL ultrapure water). Residual H_2_O_2_ concentrations were quantified by measuring the absorbance at 405 nm.

DPPH Scavenging Assay

0.1 mM DPPH radical solution (1 mL) was mixed with 1 mL nanoparticles solution (100 μg/mL) and incubated in the dark at 25°C for 60 minutes. Absorbance was measured at 517 nm using a UV-Vis spectrophotometer.

•OH Scavenging Assay

•OH were generated via a Fenton-like reaction by combining 800 μL deionized water, 20 μL H₂O₂ (3%), 100 μL 3,3′,5,5′-Tetramethylbenzidine (TMB, 2 mg/mL), and 20 μL CuCl₂ (1 mg/mL) with 20 μL nanoparticles solution (100 μg/mL). Absorbance of the oxidized TMB product was measured at 652 nm.

Cytochrome c Oxidase (CcO) Mimetic Activity

CcO (1 mg/mL in PBS, 100 μL) was mixed with 100 μL nanoparticle suspension (1 mg/mL) in 1 mL PBS (pH 7.4). After 60 minutes, nanoparticles were removed by centrifugation, and residual CcO was assessed via UV-vis spectroscopy.

NAD^+^ contents detection

stem cells were treated with or without nanozymes for 48 hours. Cells were lysed in cold buffer (1:4 dilution), and NAD^+^ levels were measured using a commercial NAD^+^/NADH assay kit (Beyotime) via the WST-8 method. For NADH quantification, lysates were heat-treated at 60°C for 30 minutes to degrade NAD^+^ prior to analysis. Samples (20 μL) were mixed with alcohol dehydrogenase, incubated at 37°C for 10 minutes, and reacted with chromogenic solution for 1 hour. Total NAD^+^/NADH content was determined by omitting the heat-treatment step. Absorbance at 450 nm was measured against a standard curve to calculate concentrations.

Intracellular ROS Scavenging

Stem cells seeded in 24-well plates were treated with nanoparticles for 24 hours, followed by exposure to 400 μM H₂O₂ for 2 hours. Cells were stained with 10 μM 2',7'-Dichlorodihydrofluorescein diacetate (DCFH-DA) for 60 minutes, washed with PBS and imaged by fluorescence microscopy. ROS levels were quantified by fluorescence intensity analysis (ImageJ 1.54 software).

**
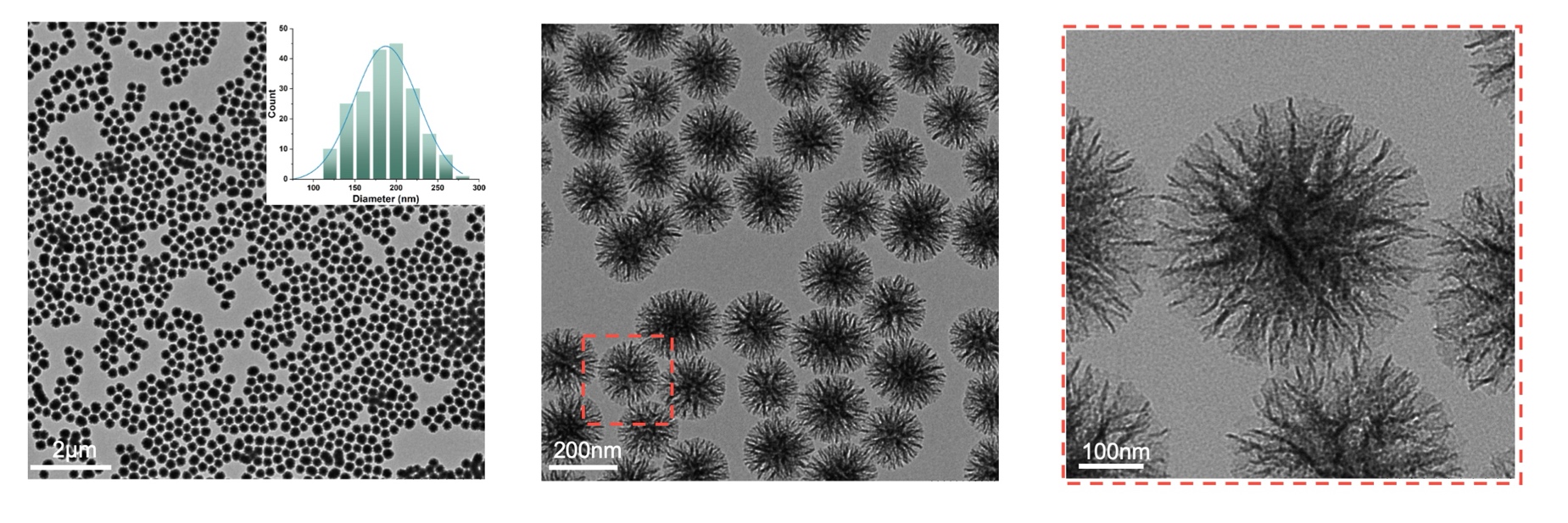
**

**Figure S1.** TEM images of uniform spherical DMSNs.


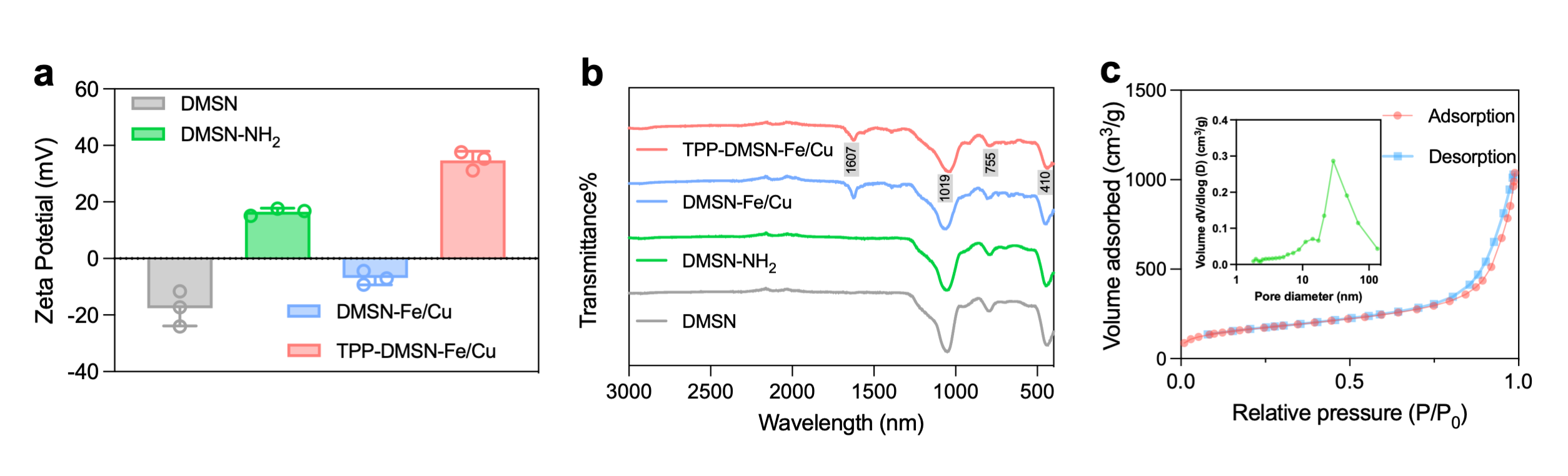


**Figure S2. a,** **b** Zeta potential values (a) and FTIR spectra (b) of different nanoparticles. **c** N_2_ adsorption and desorption isotherms and corresponding pore size distribution of TPP-DMSN-Fe/Cu nanozyme.


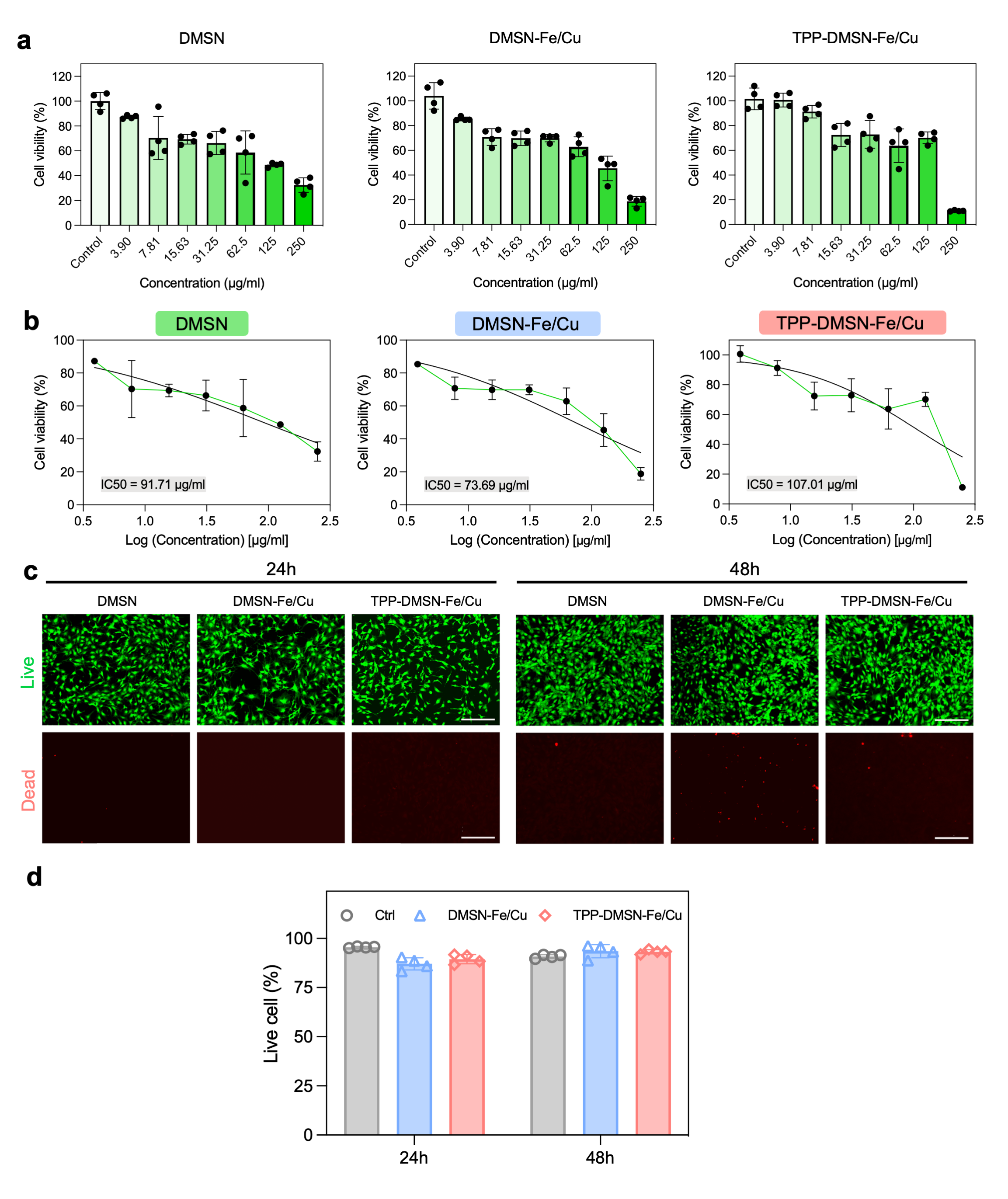


**Figure S3. a** CCK8 results of stem cells after coincubation with different concentrations of DMSN, DMSN-Fe/Cu, and TPP- DMSN-Fe/Cu for 24 hours. **b** IC_50_ results of stem cells co-cultured with different nanoparticles for 24 hours. **c, d** Live/dead staining images of stem cells co-cultured with nanozymes for 24 and 48 hours (**c**) and their corresponding fluorescence intensities (**d**). Scale bar = 300 µm. Data are presented as mean ± s.d., *n* = 4 biologically independent samples.


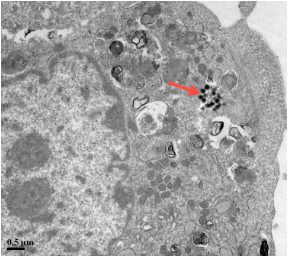


**Figure S4**. TEM images after 4 hours of treatment with DMSN-Fe/Cu nanozymes (red arrows indicate nanozymes). Scale bar = 0.5 µm.


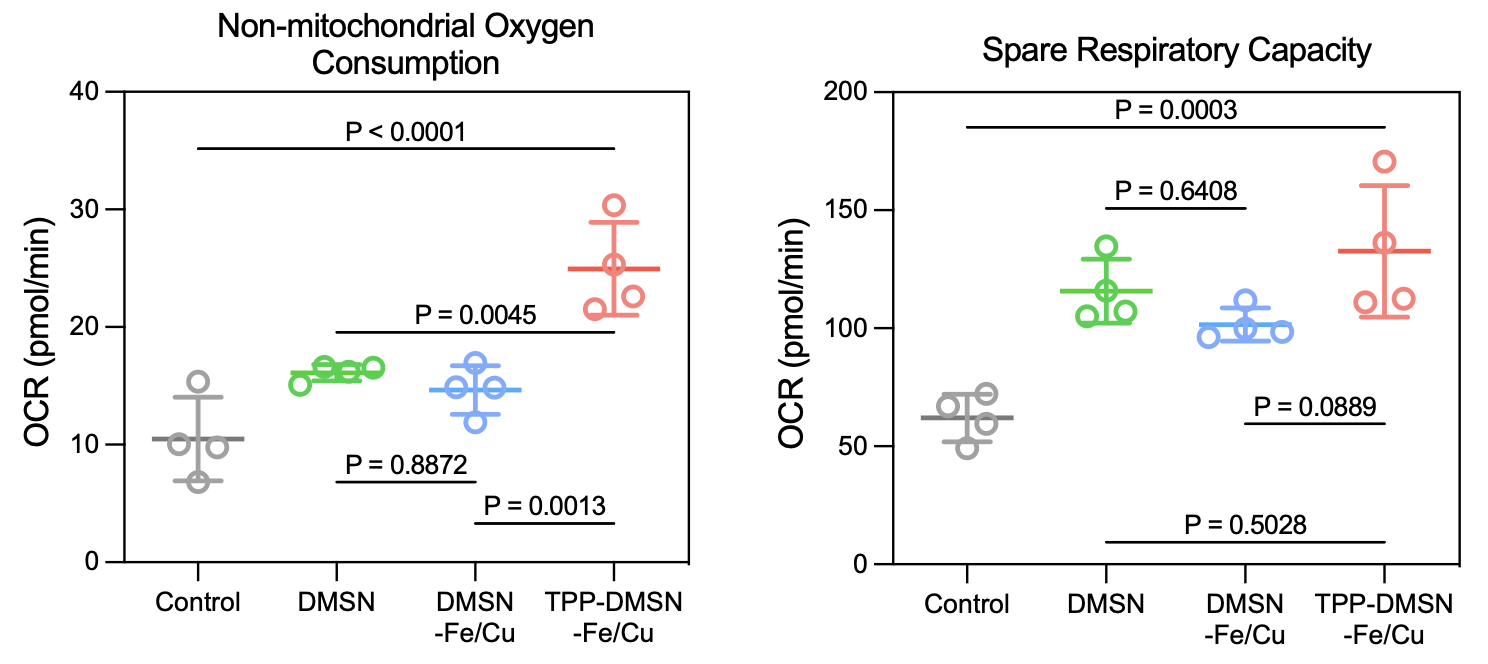


**Figure S5.** Real-time OCRs of stem cells in the cell mitochondrial stress test and semiquantitative analysis of non-mitochondrial oxygen consumption and spare respiratory capacity. Data are presented as mean ± s.d., *n* = 4 biologically independent samples, by one-way ANOVA with Tukey’s post hoc test. The *P* value is noted.

**
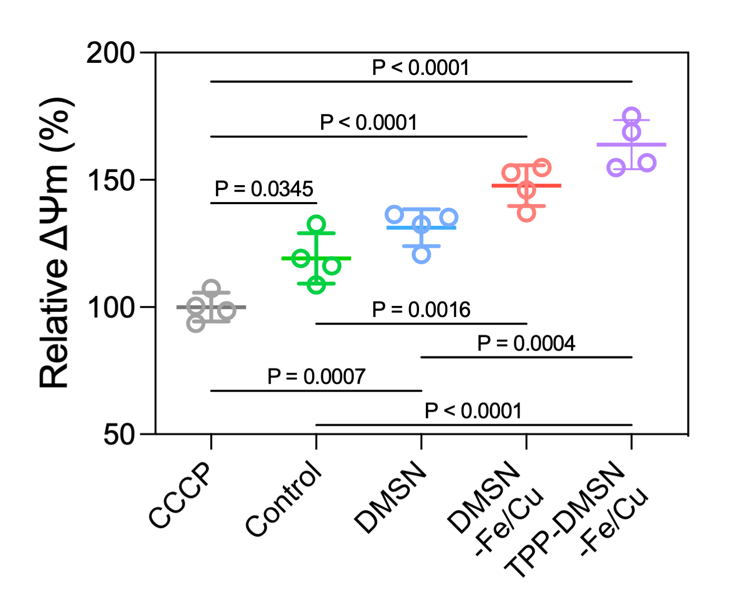
**

**Figure S6.** Quantitative analysis of JC-1 aggregates indicating mitochondrial membrane potential in cells after 7 days of osteogenic differentiation. Data are presented as mean ± s.d., *n* = 4 biologically independent samples, by one-way ANOVA with Tukey’s post hoc test. The *P* value is noted.


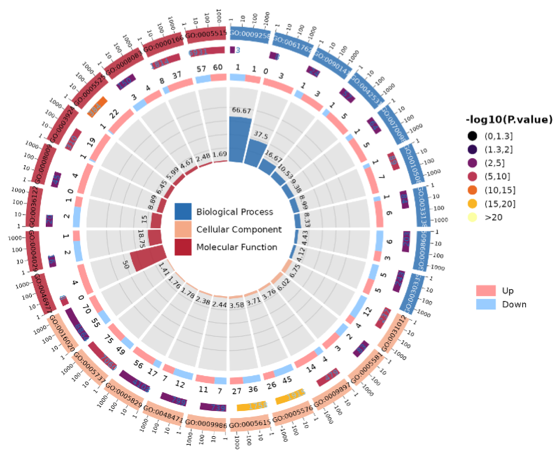


**Figure S7.** GO term multidimensional enrichment string diagram.


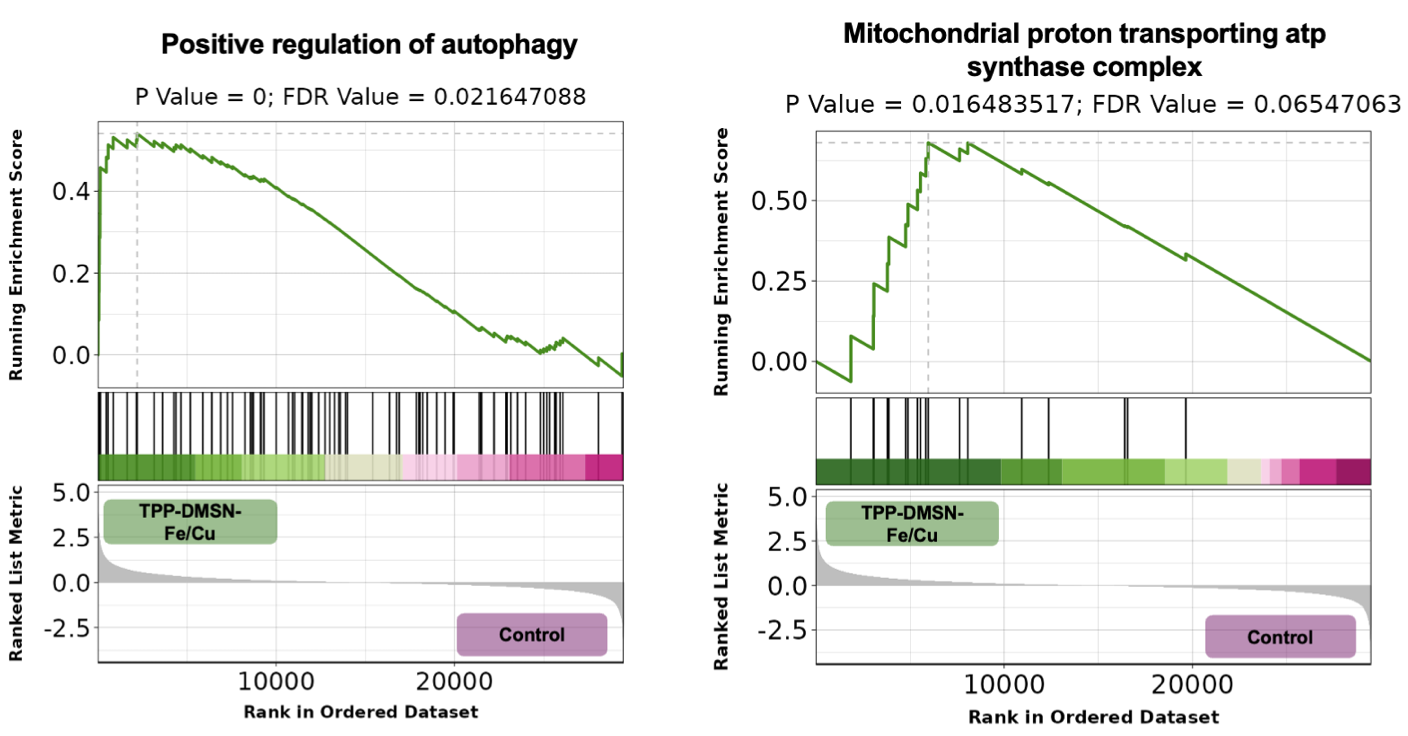


**Figure S8.** GSEA plot showing enrichment of the “Positive regulation of autophagy” and “Mitochondrial proton transporting atp synthase complex” gene set in control and TPP- DMSN-Fe/Cu groups.


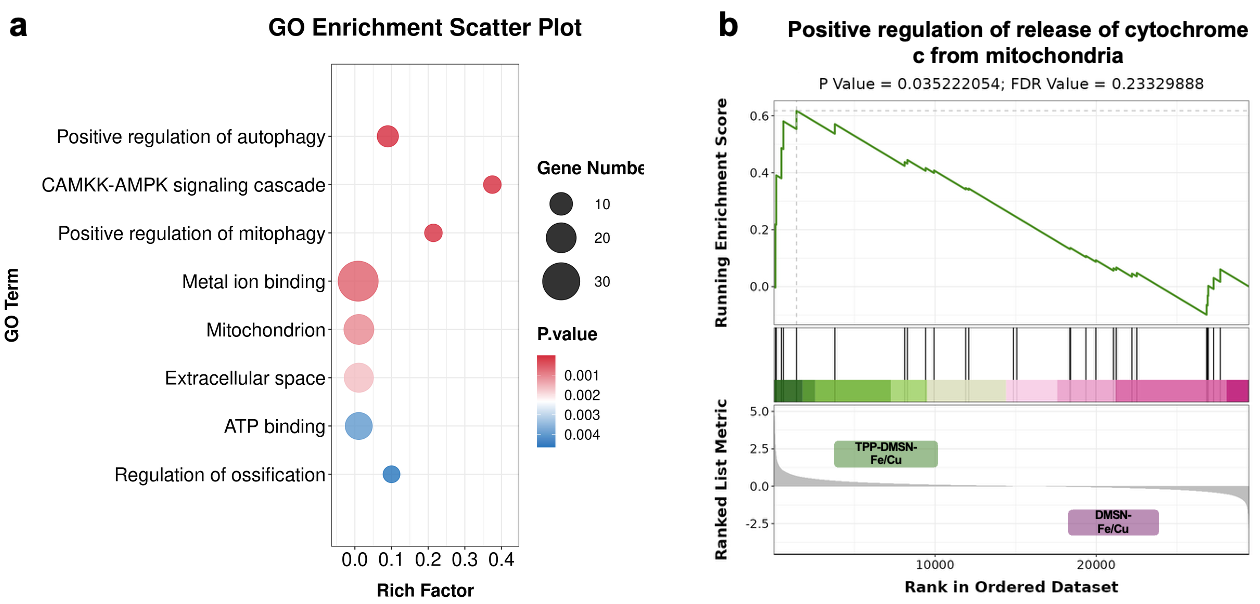


**Figure S9.** (**a**) Gene Ontology enrichment scatter plot comparing cells treated with TPP-DMSN-Fe/Cu and those with DMSN-Fe/Cu. (**b**) GSEA plot showing enrichment of the “Positive regulation of release of cytochrome c from mitochondria” gene set in TPP- DMSN-Fe/Cu and DMSN-Fe/Cu groups.


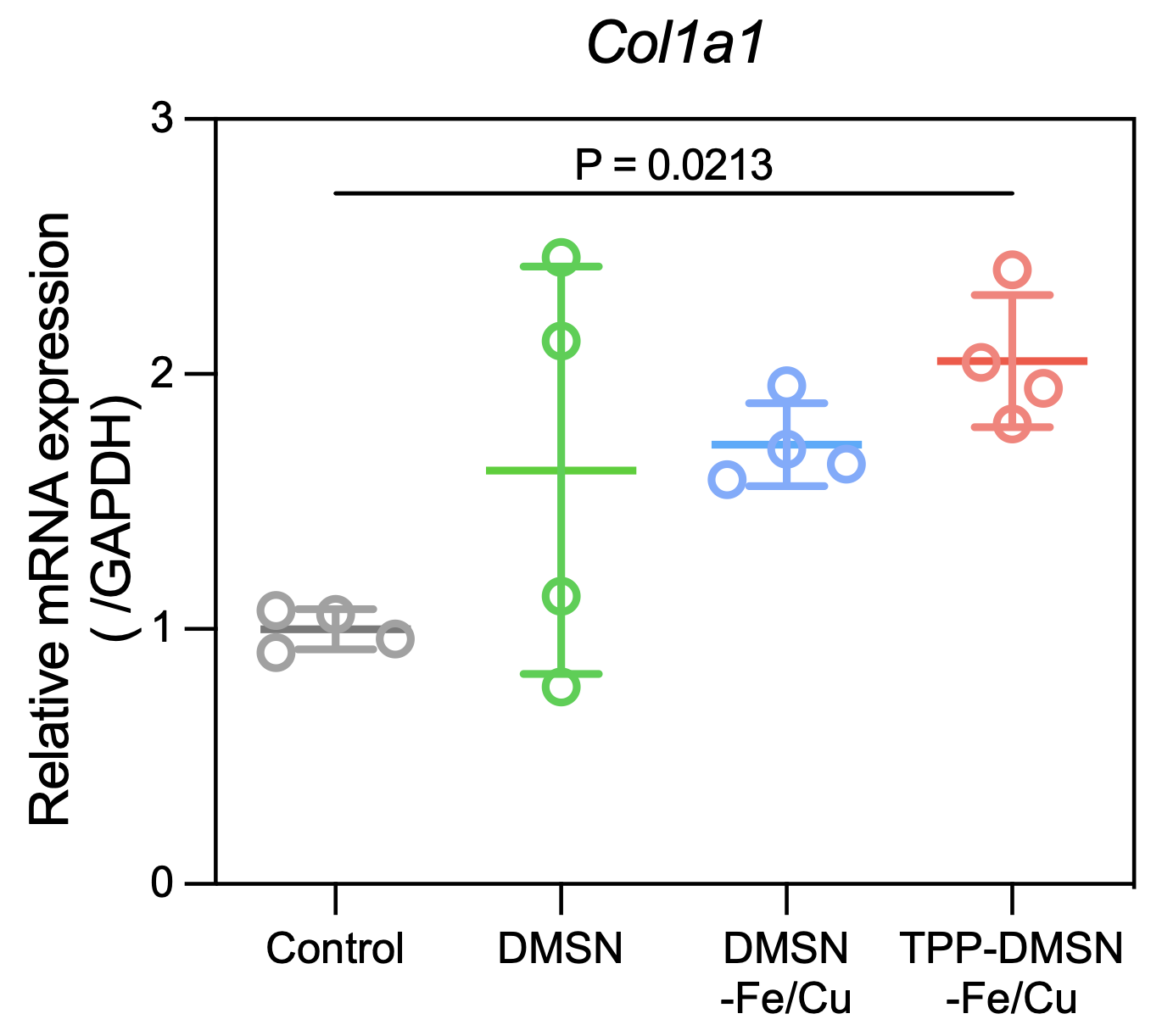


**Figure S10.** Gene expression of the osteogenic markers *Col1a1* in the stem cells. Data are presented as mean ± s.d., *n* = 4 biologically independent samples, by one-way ANOVA with Tukey’s post hoc test. The *P* value is noted.


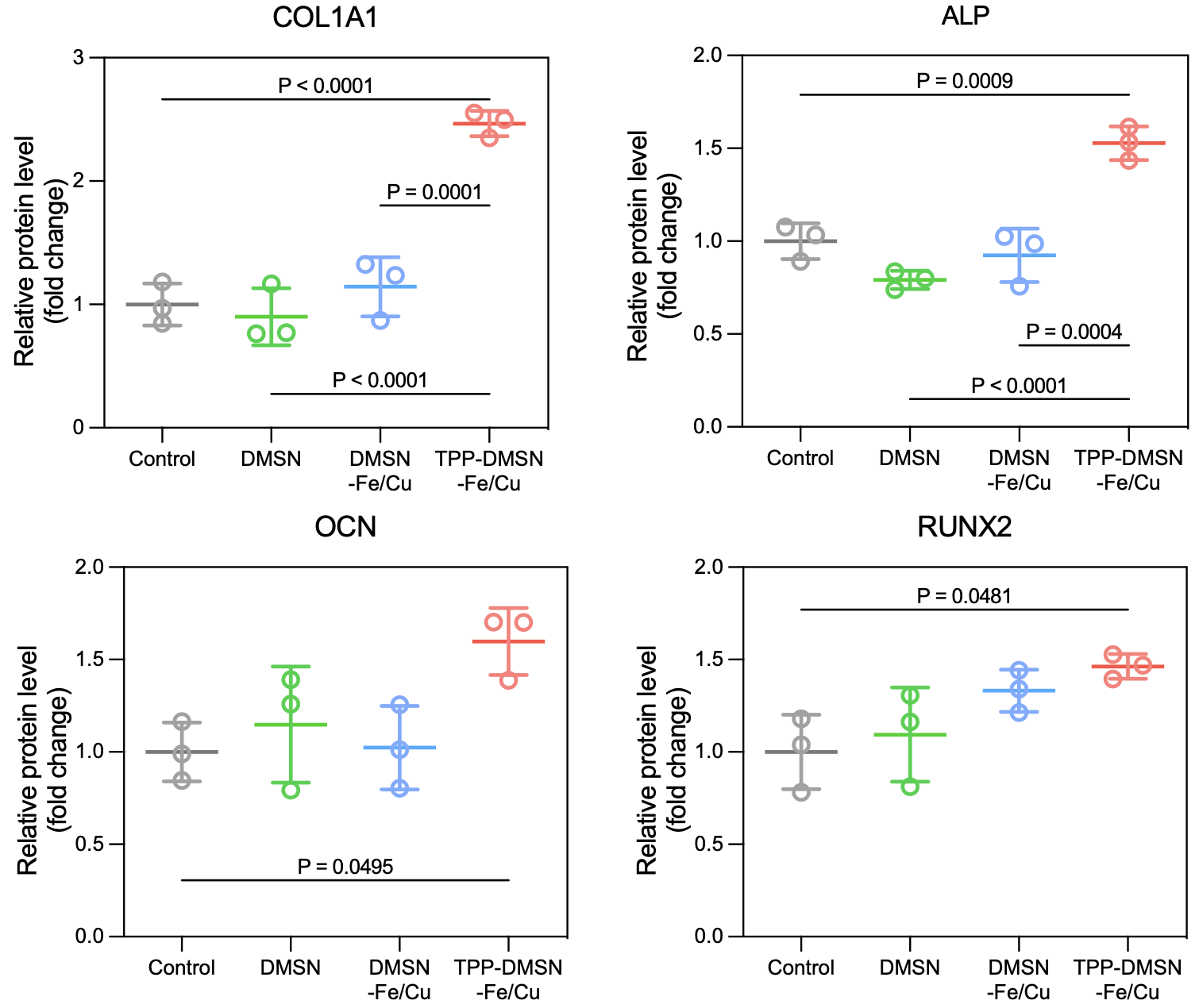


**Figure S11.** Western blot quantitative analysis of osteogenic markers: COL1A1, RUNX2, ALP, OCN, using ImageJ 1.54 software. Data are presented as mean ± s.d., *n* = 3 biologically independent samples, by one-way ANOVA with Tukey’s post hoc test. The *P* value is noted.


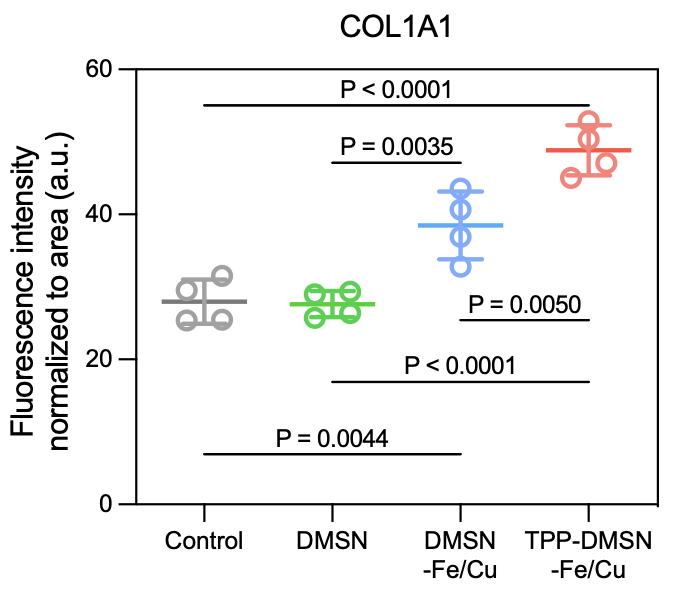

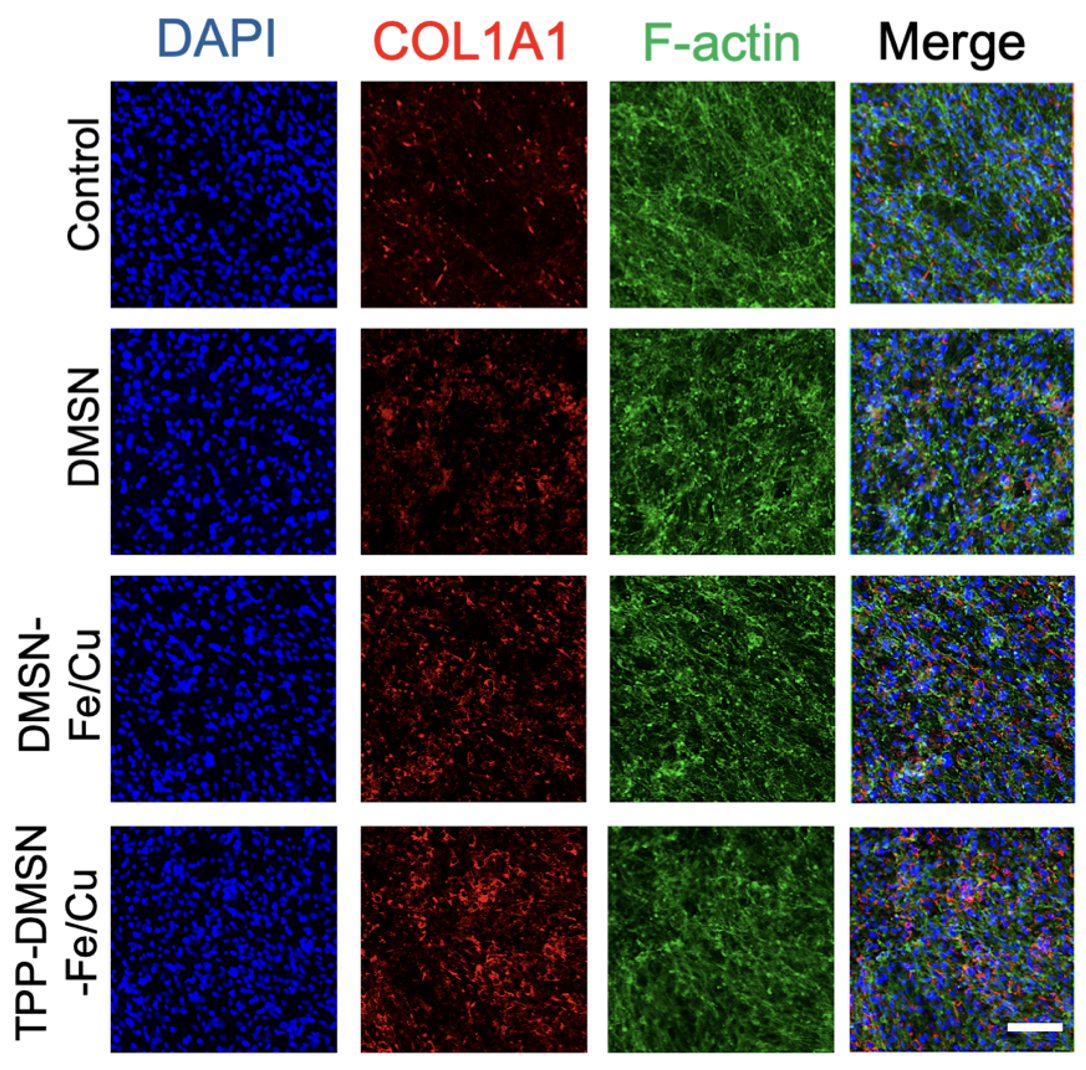


**Figure S12.** Immunofluorescence staining images of COL1A1 at 7 days. Scale bar = 100 μm. Data are presented as mean ± s.d., *n* = 4 biologically independent samples, by one-way ANOVA with Tukey’s post hoc test. The *P* value is noted.


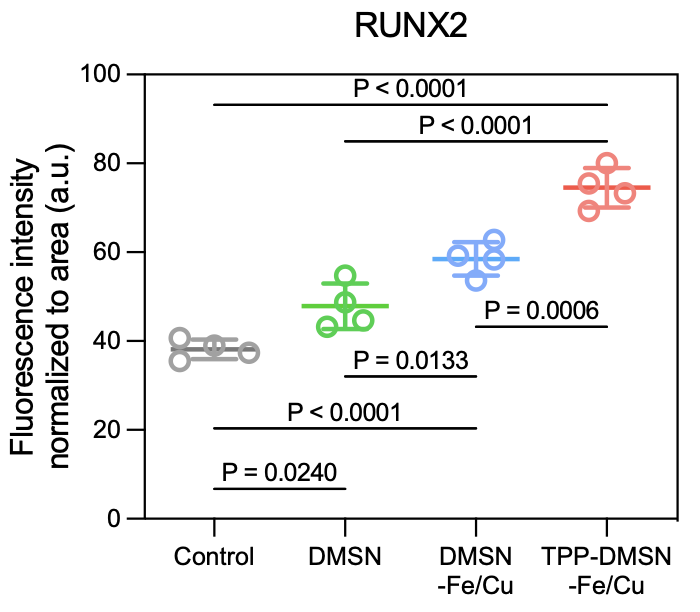

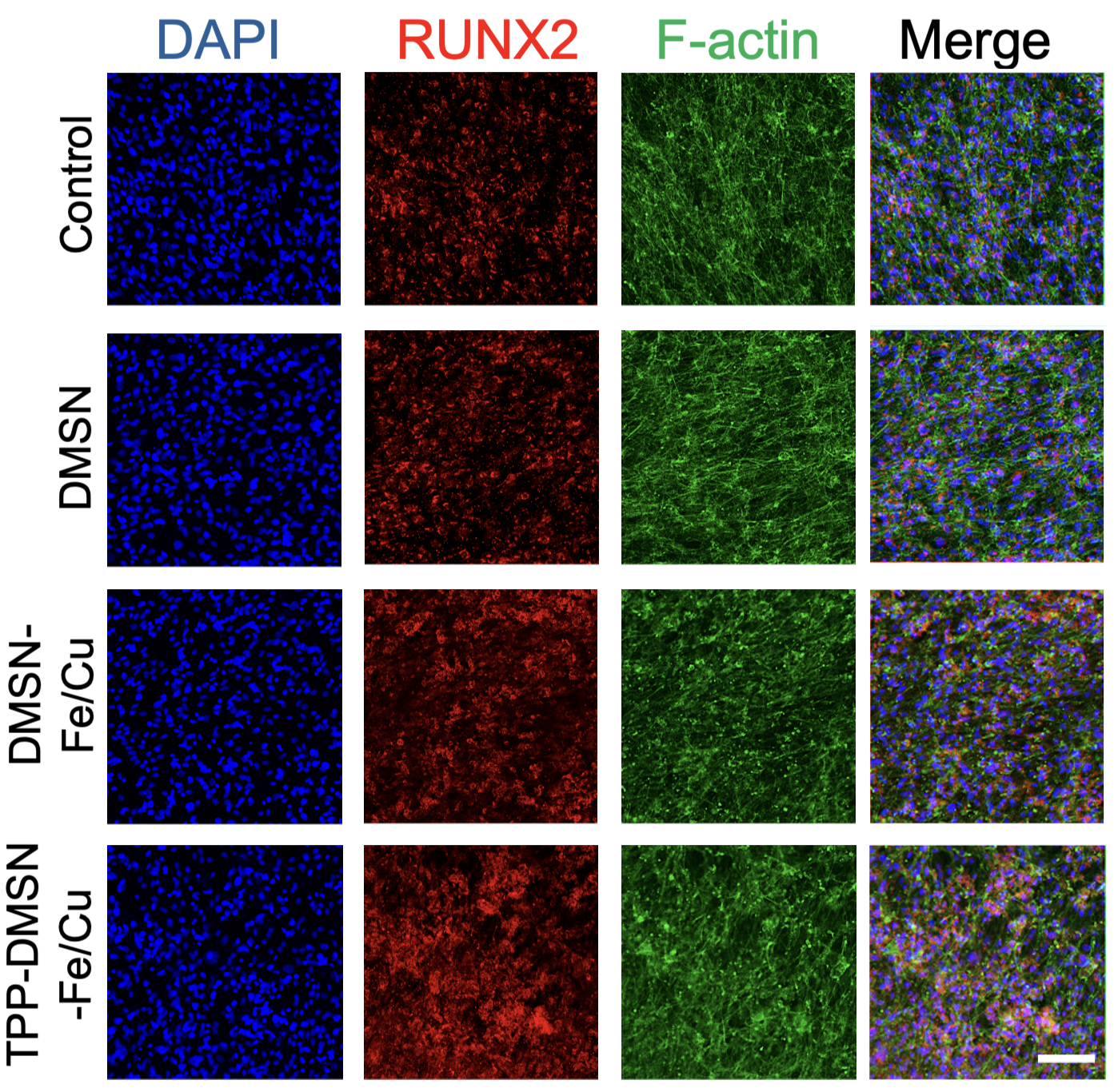


**Figure S13.** Immunofluorescence staining images of RUNX2 at 7 days. Scale bar = 100 μm. Data are presented as mean ± s.d., *n* = 4 biologically independent samples, by one-way ANOVA with Tukey’s post hoc test. The *P* value is noted.


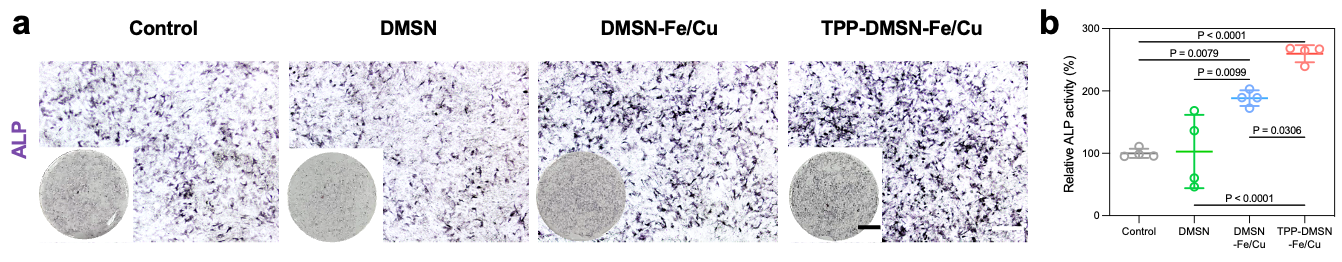


**Figure S14.** (**a**) ALP staining images for stem cells treated by different nanoparticles for 7 days. Scale bar = 2 mm (for the entire culture well) and 100 µm (for the magnified view). (**b**) Relative ALP activity of the cells. Data are presented as mean ± s.d., *n* = 4 biologically independent samples, by one-way ANOVA with Tukey’s post hoc test. The *P* value is noted.


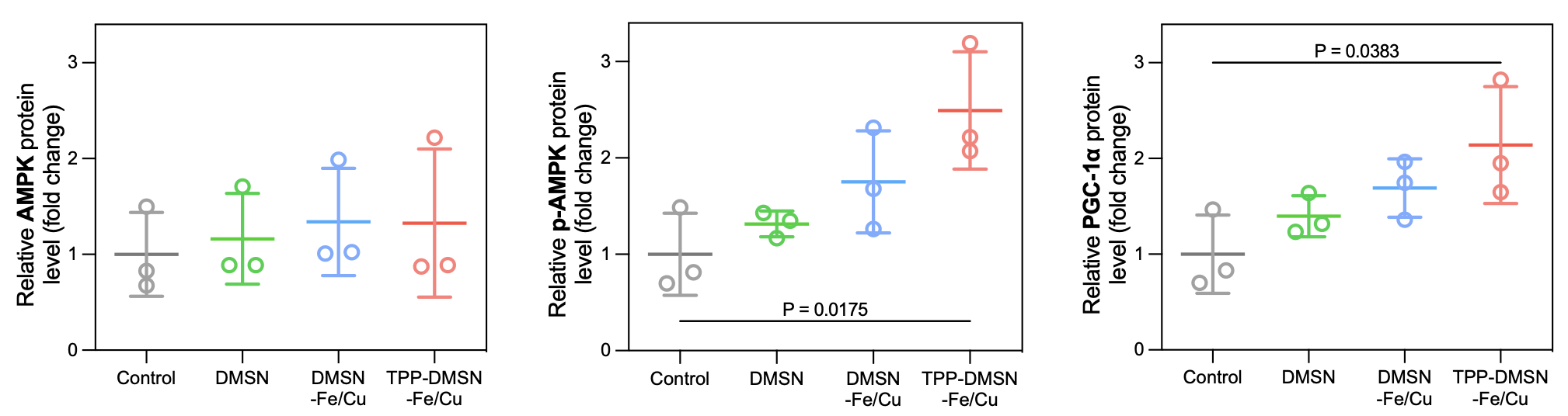


**Figure S15.** Western blot quantitative analysis of AMPK, p-AMPK, and PGC-1α, using ImageJ 1.54 software. Data are presented as mean ± s.d., *n* = 3 biologically independent samples, by one-way ANOVA with Tukey’s post hoc test. The *P* value is noted.

**
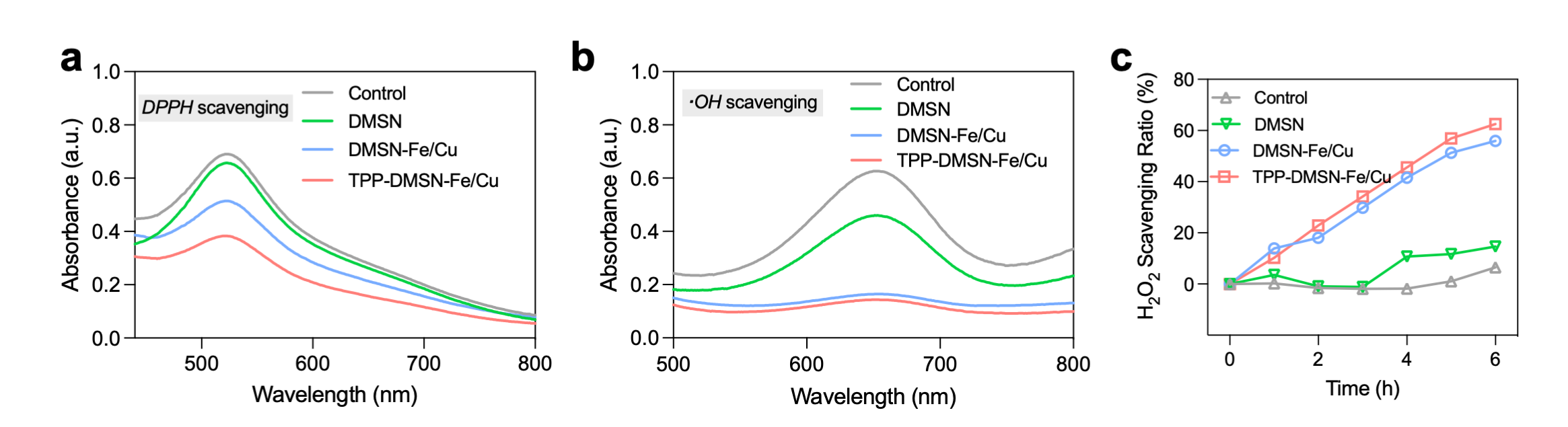
**

**Figure S16.** Antioxidant assays comparing the DPPH- (**a**), •OH- (**b**), and H₂O₂-scavenging (**c**) rates of different nanoparticles.


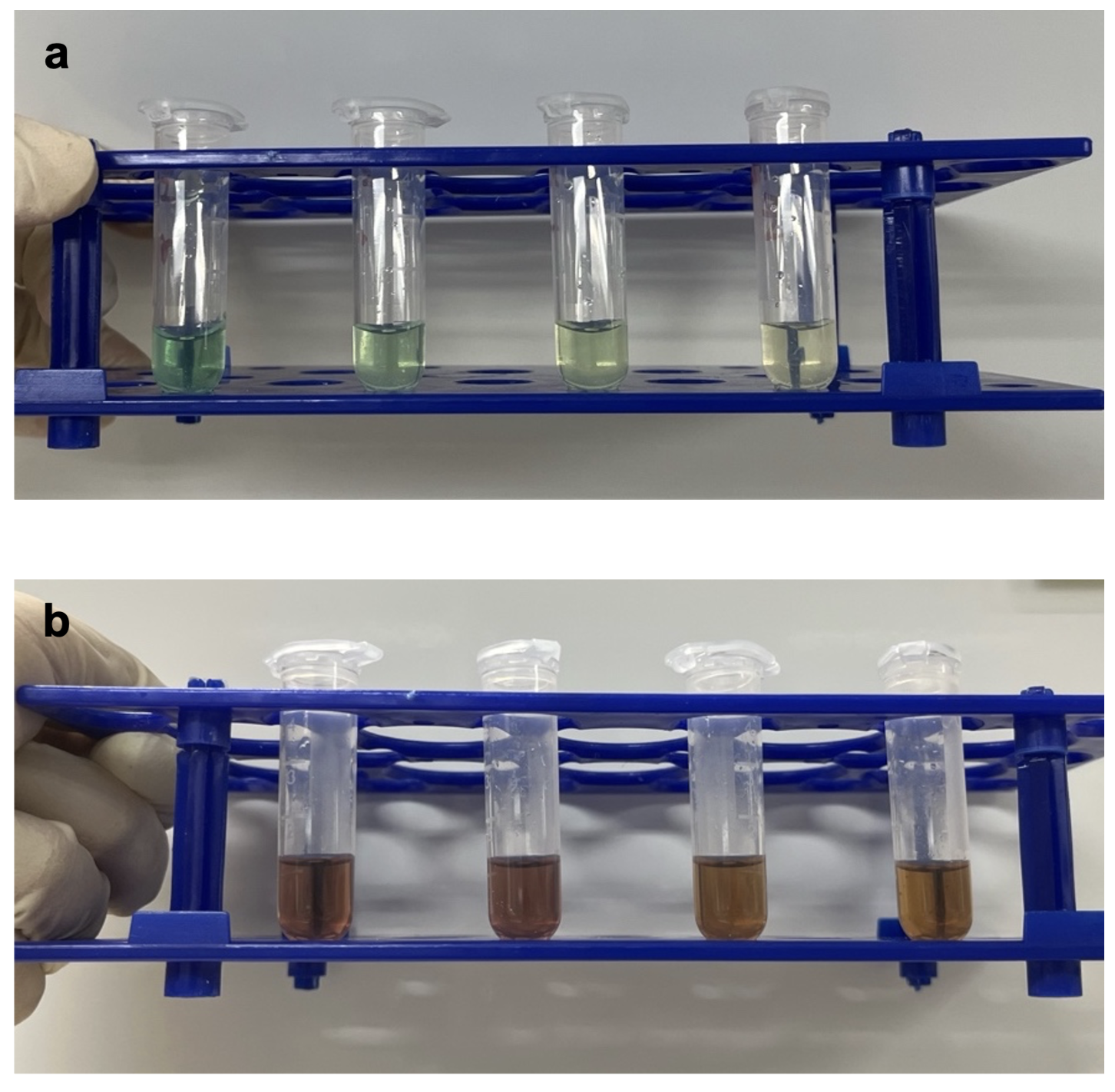


**Figure S17.** **a** Scavenging capacity of NPs for •OH. **b** Scavenging capacity of NPs for DPPH.


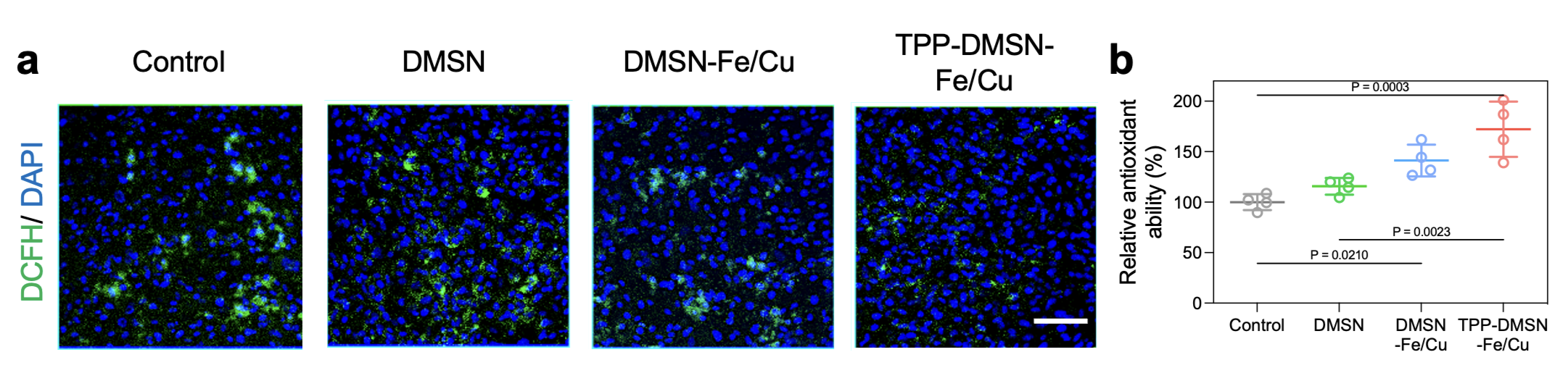


**Figure S18. a** ROS staining detected by 2′,7′-dichlorodihydrofluorescein diacetate (DCFH-DA) for 4 samples after 24h of treatment by different nanoparticles, and **b** corresponding ROS scavenging ability analysis. Scale bar = 100 µm.


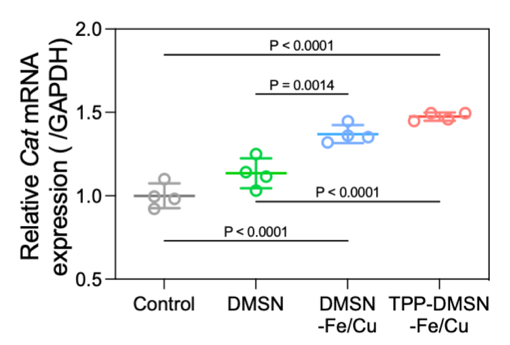


**Figure S19.** Gene expression of the *Cat* in the stem cells. Data are presented as mean ± s.d., *n* = 4 biologically independent samples, by one-way ANOVA with Tukey’s post hoc test. The *P* value is noted.


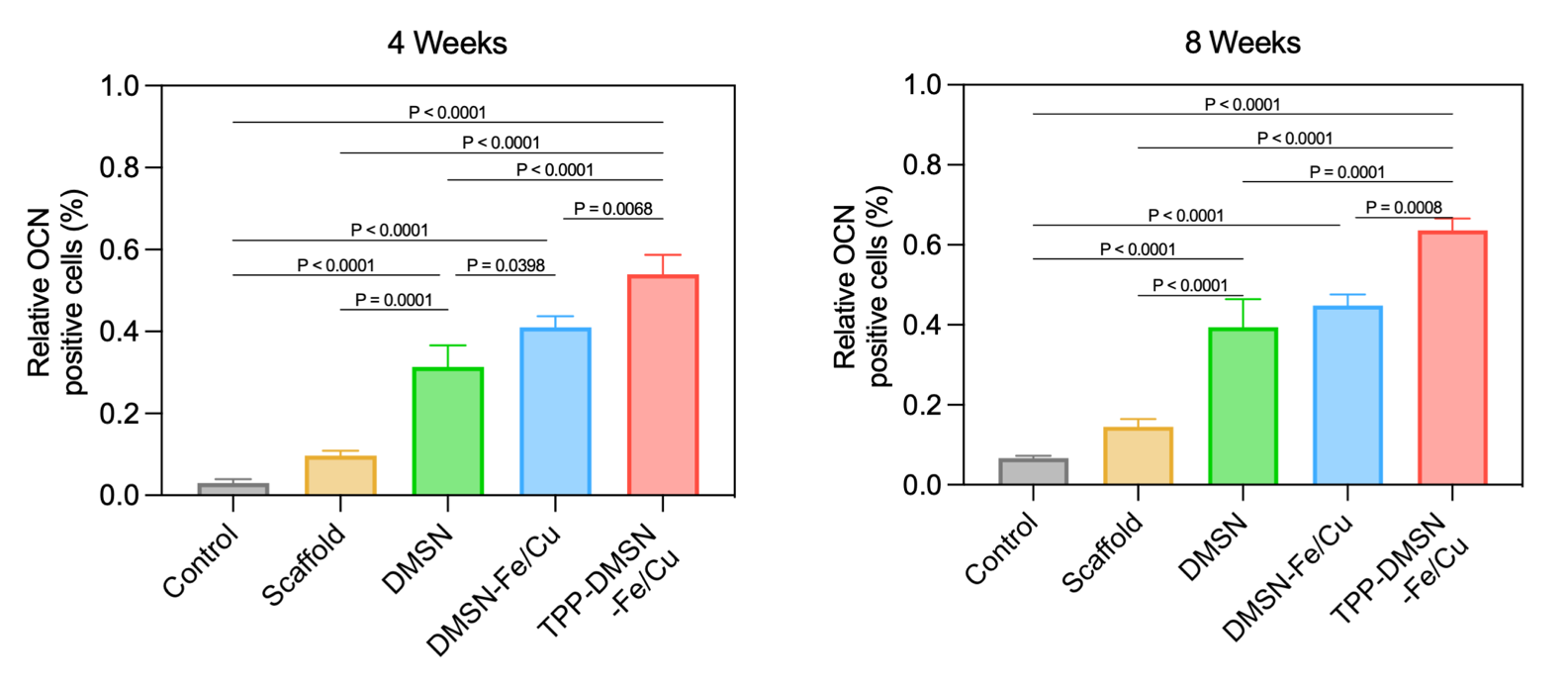


**Figure S20.** The number of OCN-positive cells in different groups. Data are presented as mean ± s.d., *n* = 3 biologically independent samples, by one-way ANOVA with Tukey’s post hoc test. The *P* value is noted.


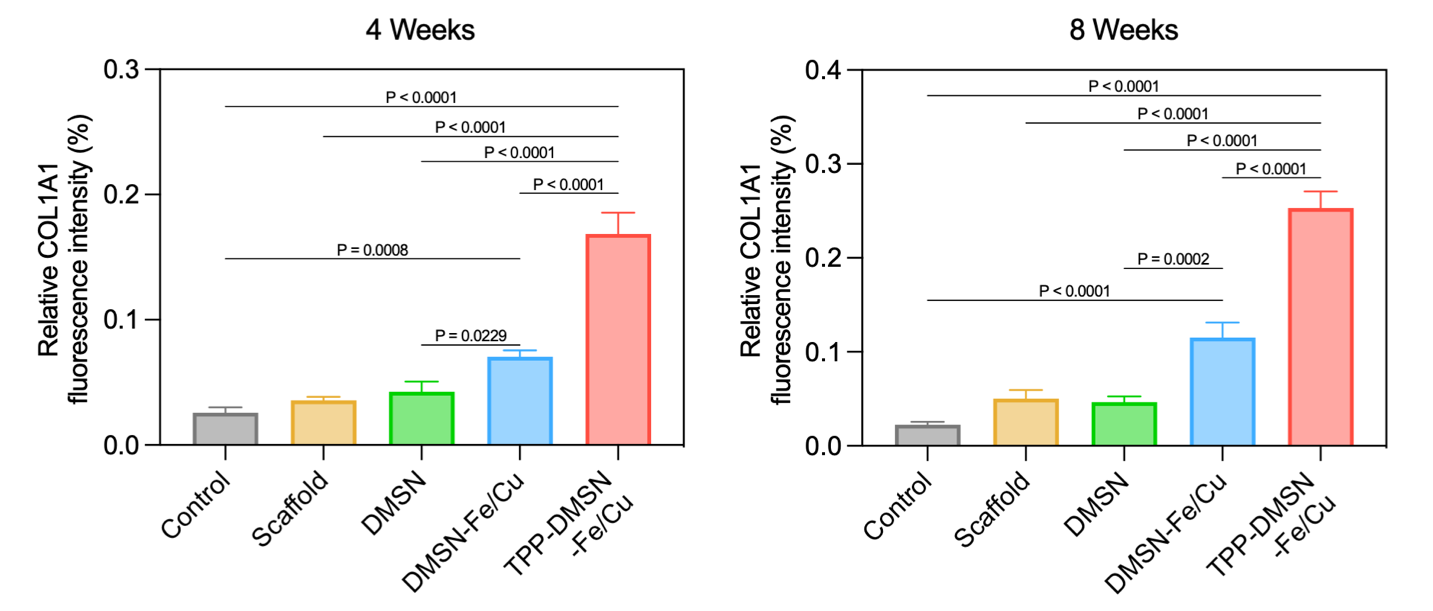


**Figure S21.** Immunofluorescence staining images intensity of COL1A1. Data are presented as mean ± s.d., *n* = 3 biologically independent samples, by one-way ANOVA with Tukey’s post hoc test. The *P* value is noted.


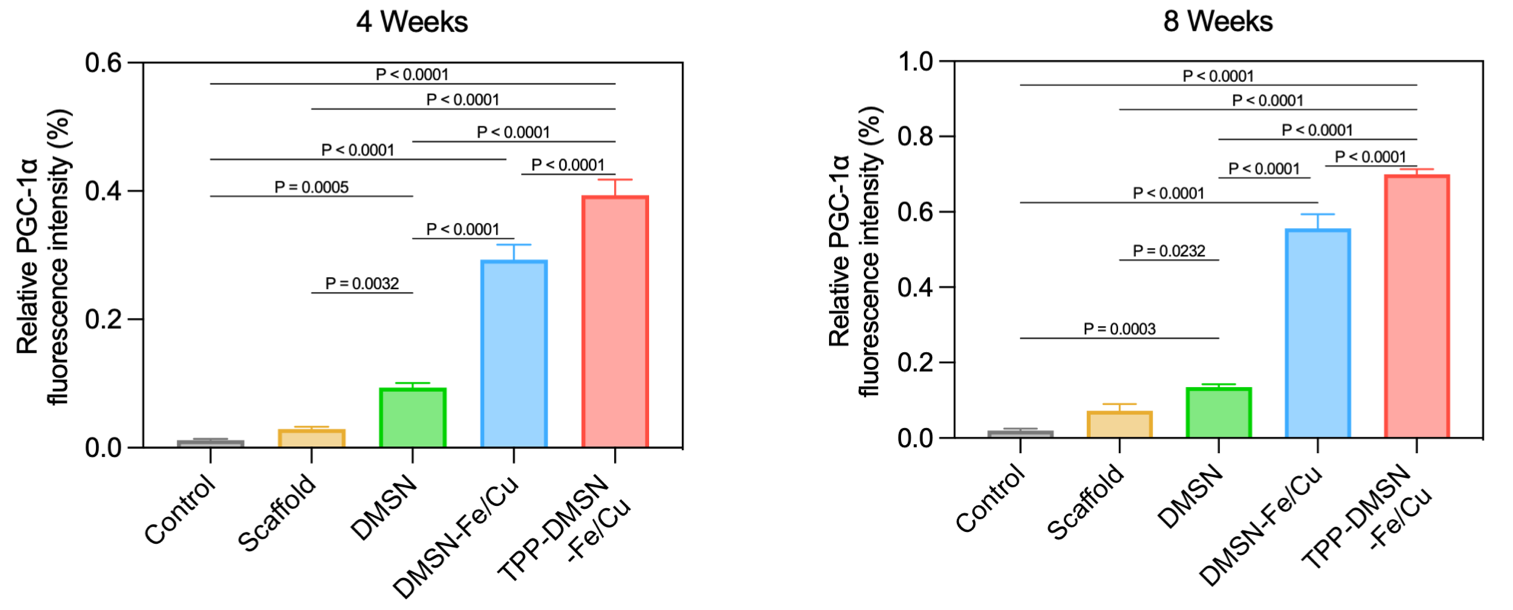


**Figure S22.** Immunofluorescence staining images intensity of PGC-1α. Data are presented as mean ± s.d., *n* = 3 biologically independent samples, by one-way ANOVA with Tukey’s post hoc test. The *P* value is noted.


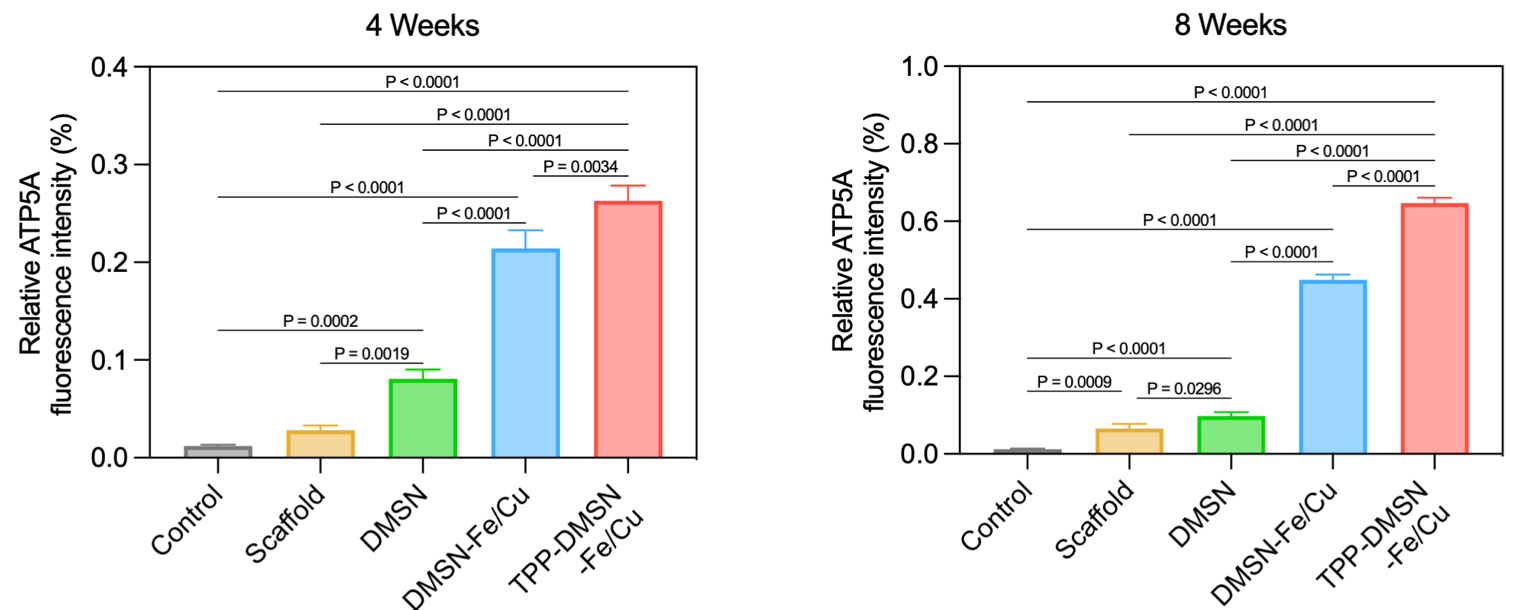


**Figure S23.** Immunofluorescence staining images intensity of ATP5A. Data are presented as mean ± s.d., *n* = 3 biologically independent samples, by one-way ANOVA with Tukey’s post hoc test. The *P* value is noted.

**
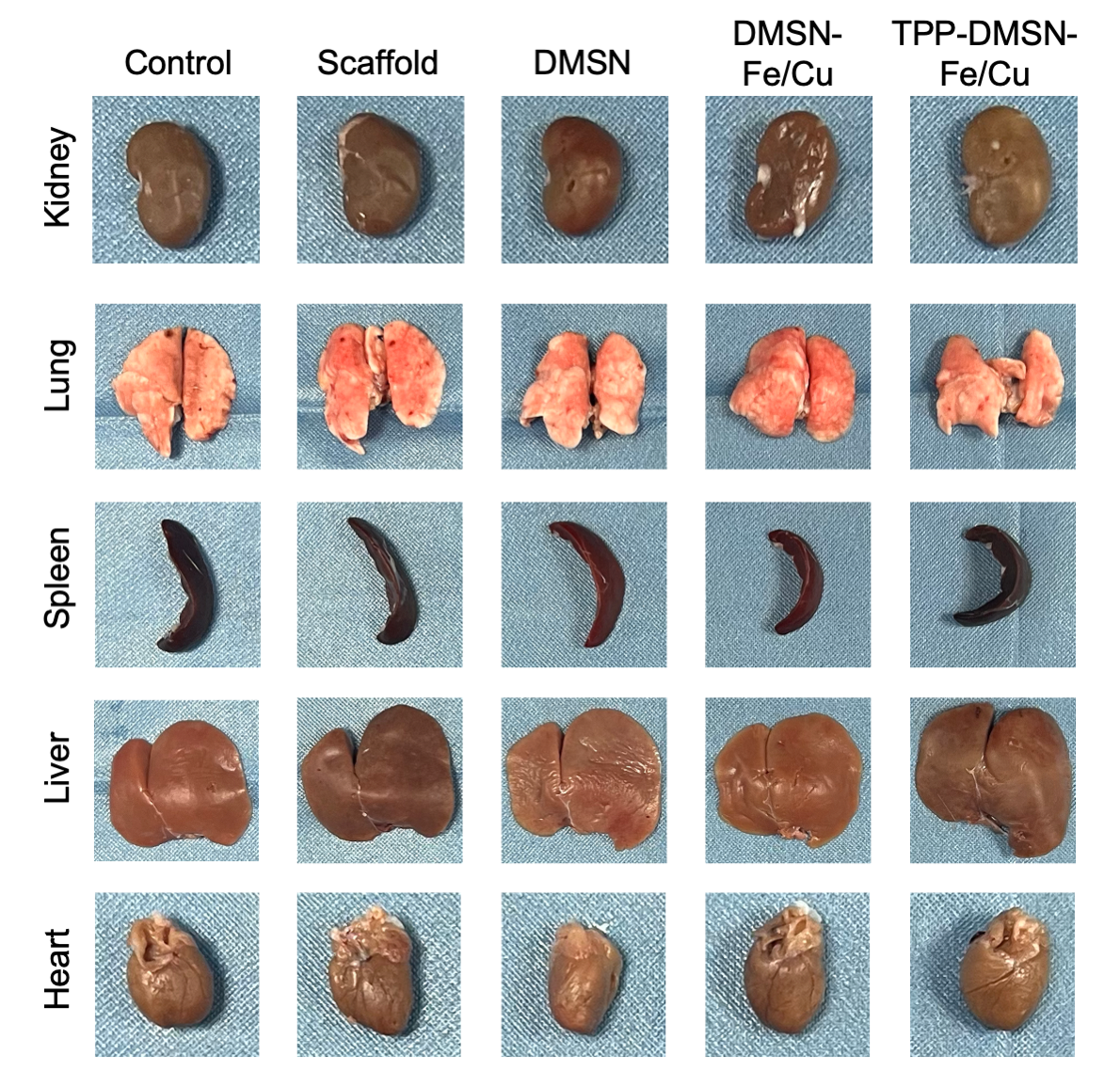
**

**Figure S24.** Biocompatibility evaluation *in vivo*. The photographs of the heart, liver, spleen, lung, and kidney.

**
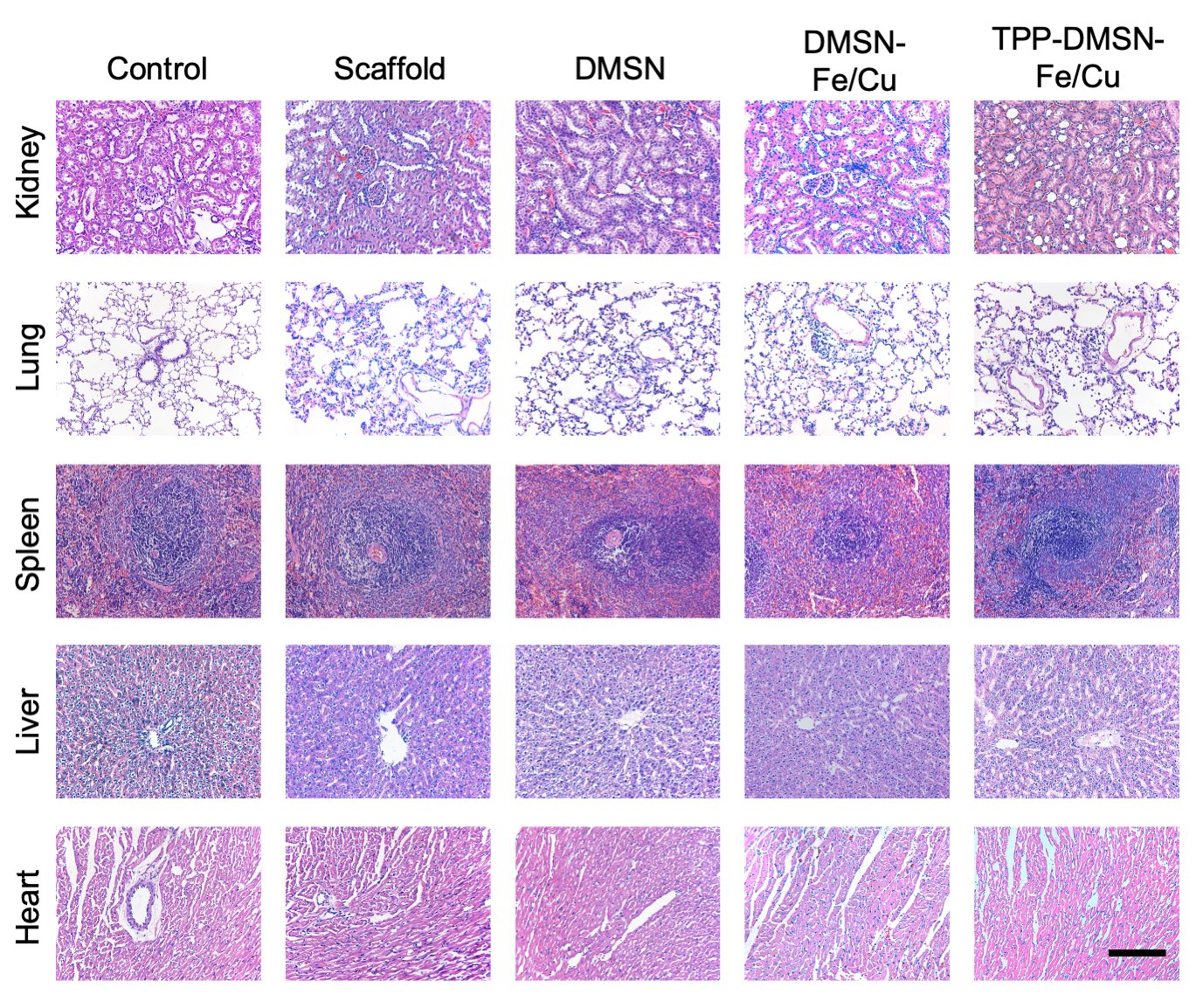
**

**Figure S25.** The tissue sections H&E staining of the heart, liver, spleen, lung, and kidney. Scale bar = 200 μm.


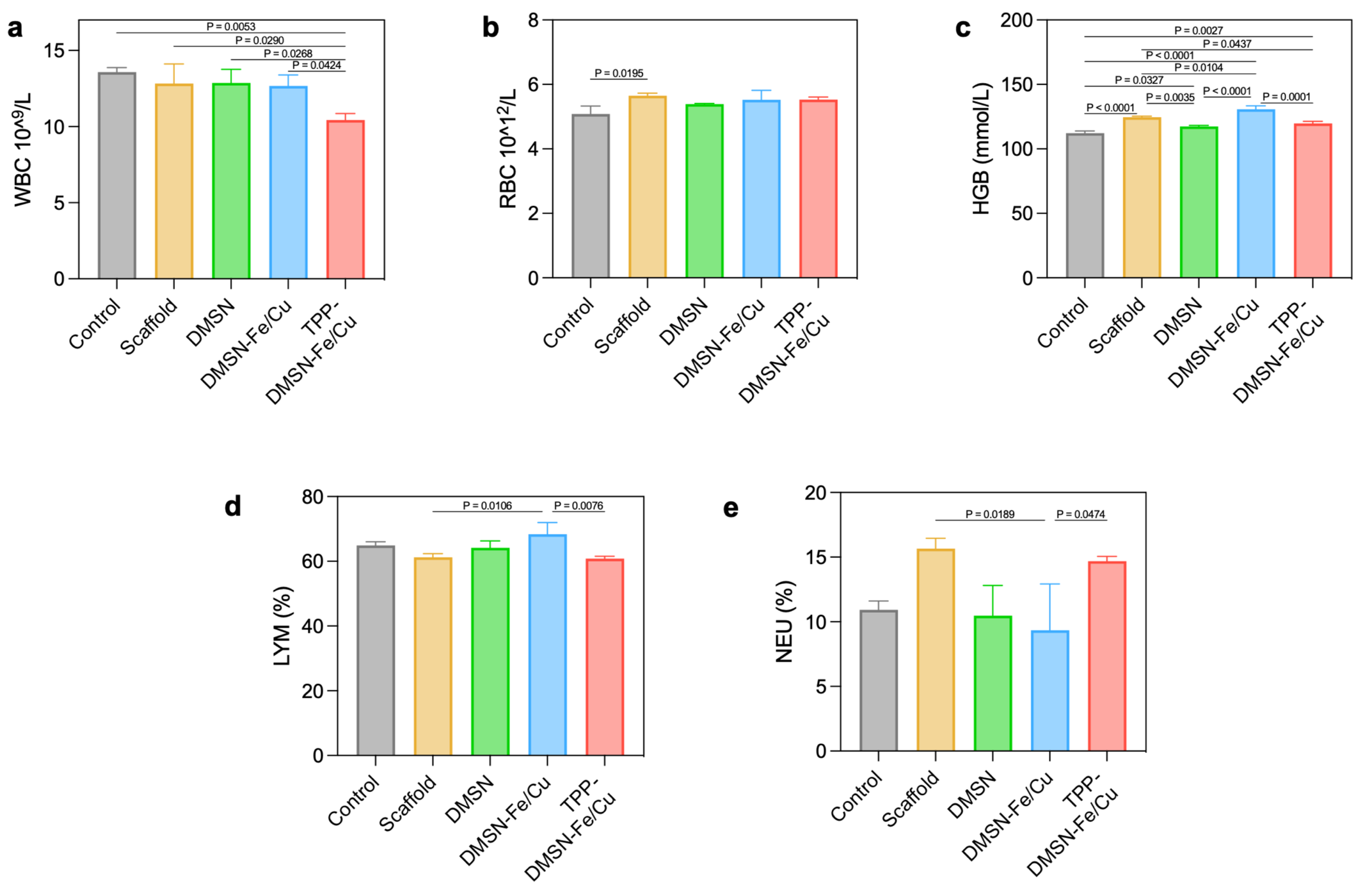


**Figure S26.** **a** Blood indices of rat femoral defect model after different treatments, including **a** white blood cell count (WBC), **b** red blood cell count (RBC), **c** hemoglobin (HGB), **d** lymphocyte percentage (Lymph %), and **e** Neutrophils percentage (NEU %). Data are presented as mean ± s.d., *n* = 3 biologically independent samples, by one-way ANOVA with Tukey’s post hoc test. The *P* value is noted.

**Table S1.** All gene primer sequences used in the RT-qPCR experiments.

|  | Gene Name | Forward Sequences | Reverse Sequences |
| --- | --- | --- | --- |
| Osteogenic | *Runx2* | GGTGAAACTCTTGCCTCGTC | AGTCCCAACTTCCTGTGCT |
|  | *Col1a1* | GCTCCTCTTAGGGGCCACT | ATTGGGGACCCTTAGGCCAT |
|  | *Ocn* | TCTGACAAAGCCTTCATGTCC | AAATAGTGATACCGTAGATGCG |
|  | *Opn* | ACACTTTCACTCCAATCGTCC | TGCCCTTTCCGTTGTTGTCC |
|  | *Alp* | GGCTGGAGATGGACAAATTCC | CCGAGTGGTAGTCACAATGCC |
| Metabolism | *Opa1* | CTGCAGGTCCCAAATTGGTT | TCTTTGTCTGACACCTTCCTGT |
|  | *Cpt1a* | TCGGTGAGCCTGGCCT | TTGAGTGGTGACCGAGTCTG |
|  | *Drp1* | CCAGAGGAACTGGTGTGGTC | CCATTCTTCTGCTTCAACTCCATT |
|  | *Fam36a* | GAAAAAGCCCTTCAAGCTCCT | GAAATGTCCAAGTCCAGTCACA |
|  | *Atp5a* | TCTCCATGCCTCTAACACTCG | CCAGGTCAACAGACGTGTCAG |
|  | *Sdha* | GGAACACTCCAAAAACAGACCT | CCACCACTGGGTATTGAGTAGAA |
|  | *Cat* | ATGGTCACCGGCACATGAAT | GCCCTGGTCGGTCTTGTAAT |
|  | *Pgc-1a* | TATGGAGTGACATAGAGTGTGCT | GTCGCTACACCACTTCAATCC |
|  | GAPDH | AGGTCGGTGTGAACGGATTTG | GGGGTCGTTGATGGCAACA |
|  |  |  |  |

**Table S2**. Information of antibodies used in this western blot experiment.

| **Antibody**  **Reference** | **Company, Catalog** | **Dilution** |
| --- | --- | --- |
| GAPDH | Abcam, ab8245 | 1:2000 |
|  |  |  |
| **Osteogenic marker** |  |  |
| Anti-Collagen I | Abcam, ab270993 | 1:1000 |
| Anti-RUNX2 | Abcam, ab236639 | 1:1000 |
| Anti-ALP | Abcam, ab229126 | 1:1000 |
| Anti-Osteocalcin | Abcam, ab309521 | 1:1000 |
|  |  |  |
| **Signal pathway marker** |  |  |
| Anti-Beclin1 | Abcam, ab207612 | 1:2000 |
| Anti-LC3B | Abcam, ab192890 | 1:2000 |

**Table S3**. Information of antibodies used in immunocytochemistry (ICC), immunohistochemistry (IHC), & tissue immunofluorescence staining experiment.

| **Antibody** | **Company, Catalog** | **Dilution** |
| --- | --- | --- |
| **For ICC** |  |  |
| Anti-Collagen I | Abcam, ab270993 | 1:200 |
| Anti-RUNX2 | Abcam, ab236639 | 1:200 |
| Anti-Osteocalcin | Abcam, ab309521 | 1:200 |
|  |  |  |
| **For IHC** |  |  |
| Anti-Osteocalcin  **For Immunofluorescence** | Abcam, ab309521 | 1:500 |
| PGC-1α | Proteintech, 66369-1-ig | 1: 500 |
| ATP-5A | Proteintech, 14676-1-AP | 1: 500 |
| COL1A1 | Affinity, AF7001 | 1: 500 |
